# Spontaneous Trait Inferences From Behavior: A Systematic Meta-Analysis

**DOI:** 10.1177/01461672221100336

**Published:** 2022-06-24

**Authors:** Antonia Bott, Larissa Brockmann, Ivo Denneberg, Espen Henken, Niclas Kuper, Felix Kruse, Juliane Degner

**Affiliations:** 1 Universität Hamburg, Germany; 2 Universität Bielefeld, Germany

**Keywords:** spontaneous trait inferences, meta-analysis, impression formation, person perception

## Abstract

Research suggests that people spontaneously infer traits from behavioral information, thus forming impressions of actors’ personalities. Such spontaneous trait inferences (STI) have been examined in a wide range of studies in the last four decades. Here, we provide the first systematic meta-analysis of this vast literature. We included data from *k* = 86 publications, with overall *N* = 13,630 participants. The average STI effect was moderate to large (*d*_z_ = 0.59) and showed substantial heterogeneity. The type of experimental paradigm significantly moderated the STI effect size, with larger effects in long-term memory–based paradigms compared with working memory–based paradigms. Generally, STI effects were robust to various methodological variations and also to potential concerns of publication bias. Contrary to expectations, cultural background (independent vs. interdependent) did not emerge as a significant moderator of STI effects. We discuss these findings with respect to their theoretical relevance and derive implications for future research and theorizing.

Theorizing and research on person perception has for a long time worked with the assumption that people rapidly form impressions of other people by drawing various inferences based on their appearances and behaviors (e.g., Uleman et al., 2008). *Spontaneous trait inferences* (STI) are one prominent and well-established phenomenon in this domain, documenting that people spontaneously infer personality traits from others’ behaviors (Uleman, Newman, & Moskowitz, 1996). For instance, upon observing or reading about a person who carries an old woman’s groceries across the street, people tend to infer that this person *is helpful* (Winter & Uleman, 1984).

A wealth of studies has examined STIs, investigating their underlying mechanisms, process characteristics, moderators, and boundary conditions in studies with a multitude of experimental designs and in various samples across different countries. Several narrative reviews have summarized this literature (e.g., Hamilton & Stroessner, 2021; Moskowitz, 2005; Orghian et al., 2015; Uleman, 1987; Uleman et al., 2005, 2012, 2008; Uleman, Newman, & Moskowitz, 1996; Uleman & Saribay, 2018). Surprisingly, however, no quantitative review or systematic meta-analysis on STIs exists to date. Given the remarkable number of studies on this topic and the phenomenon’s assumed high robustness (Uleman et al., 2008), quantifying the average overall effect size of STIs appears to be overdue. Moreover, the large variety of experimental paradigms, stimuli, and types of samples in STI research calls for moderator analyses to systematically explore which conditions affect the occurrence and size of the STI effect. Our meta-analysis fills this gap by systematically analyzing the average STI effect size, its robustness and heterogeneity, and its potentially relevant moderators. We also believe that this meta-analysis will facilitate important theoretical advances in the field of STIs and help identify open questions for future research. Furthermore, it will provide valuable information for STI researchers who seek to calibrate their experimental designs to their research endeavors. We provide a short overview of potentially relevant moderator variables in the introduction to this meta-analysis and later discuss in more detail their meaning and implications for our theorizing on STIs in impression formation.

## Experimental Paradigms for the Investigation of STIs

The focus of STI research in the past lay mostly on investigating the assumed non-intentionality and spontaneity of person inferences from behavior. This objective was initially faced with the difficulty of developing appropriate assessment techniques (Uleman et al., 2008), but a number of *indirect* measurement procedures have since been proposed (see Supplemental Material A for a comprehensive overview). STI paradigms typically require participants to process trait-implying behavioral information about actors during an initial encoding phase (e.g., to read the statement “*The reporter steps on his girlfriend’s feet as they foxtrot.”*; Winter & Uleman, 1984). In a subsequent indirect test, which often requires some sort of recognition or memory retrieval, participants are prompted with the previously implied trait words (e.g., *clumsy*) versus control words to examine whether task performance is enhanced or impaired in a way consistent with previous spontaneous person inferences (for an exception, see Orghian et al., 2017). Using these paradigms, research over the last four decades has provided tremendous empirical support for the assumption that STIs are indeed person-specific inferences that spontaneously emerge during the encoding of actor and behavior information, even without the observer’s explicit intention or subjective awareness of this process (Orghian et al., 2015; Uleman et al., 2008; Uleman, Newman, & Moskowitz, 1996).

The procedural characteristics of these various experimental paradigms offer different yet complementary advantages but also methodological restrictions for the investigation of STIs that need to be considered when interpreting their outcomes (see overview in Table 1 and more detailed description in the Supplemental Material A). Systematically analyzing effect size differences between experimental paradigms could provide valuable insights from a methodological as well as a theoretical standpoint. First, and most obviously, observing significant STI effects in *all* paradigms would support the repeatedly stated robustness of the effect (Uleman et al., 2008). Second, investigating effect size differences between paradigms provides an informative empirical basis for choosing methodological procedures in future research. Third, and most importantly, a closer inspection of the individual paradigms may allow us to cluster them based on shared methodological characteristics that, in turn, allow further conclusions with regard to the underlying mechanisms of STIs.

**Table 1.** Experimental Paradigms in STI Research.

Paradigm	Short procedural description	DV(s)	Actor-trait link	STI effect on task performance
Long-term memory	Encoding and test phase are separated (block-wise).			
Cued recall (Winter & Uleman, 1984)	Participants read trait-implying behavioral statements during encoding. In the test phase, they are provided with implied-trait and control cues (e.g., semantic cue) and are asked to recall the corresponding behavioral sentences.	Sentence recall (rate)	No	Null effect
Savings in relearning (Carlston & Skowronski, 1994)	Participants are presented with actor photos paired with trait-implying behavioral statements during encoding. In a (re-) learning phase, the same actor photos are paired with a trait word that was either implied or not implied during encoding. In the test phase, the actor photos are shown again, and participants are asked to recall the paired trait word.	Trait recall (rate)	Yes	Improvement
False recognition (Todorov & Uleman, 2002)	Participants are exposed to actor photos paired with behavioral statements that either imply or explicitly contain a trait word during encoding. In the recognition phase, each actor photo is paired with a trait word and participants are asked to indicate if the same trait word had occurred along with the same actor photo during encoding.	Error rates, RT	Yes	Impairment
Lexical decision LTM-variant (e.g., Na & Kitayama, 2011)	Participants are exposed to actor photos paired with trait-implying behavioral statements during encoding. In the test phase, they are primed with the actor photos and categorize strings of letters as words versus non-words (i.e., lexical decisions). The words either consist of traits implied by the behavioral statements or control words (e.g., novel traits).	Error rates, RT	Yes	Improvement
Working memory	The test phase follows immediately after encoding (trial-by-trial).			
Probe recognition (e.g., Ham & Vonk, 2003)	Participants read trait-implying behavioral statements which are immediately followed by the implied trait and other probe words (e.g., novel traits, verbs from the previous sentence). Their task is to decide whether the probe word was part of the previous sentence or not.	Error rates, RT	No	Impairment
Word recognition (e.g., Fiedler & Schenck, 2001)	Participants are presented with trait-implying visual behavioral information (e.g., video or monochrome silhouette pictures) during encoding. In the test phase, they see a black textbox that gradually dissolves to unveil a trait word that was either implied or not implied by the behavior. Participants are instructed to press a key as soon as they recognize the word and then report it.	RT	No	Improvement
Lexical decision WM-variant (e.g., Saribay et al., 2012)	Participants read trait-implying behavioral information during encoding. Lexical decisions are assessed immediately after the presentation of each behavioral statement.	Error rates, RT	No	Improvement
Modified free association (Orghian et al., 2017)	Participants memorize trait-implying behavioral statements and respond to probe words that are semantically related versus unrelated to the implied trait.	RT	No	Improvement

*Note.* STI = spontaneous trait inferences; DV = dependent variable; LTM = long-term memory; WM = working memory; RT = response times.

Based on one predominant characteristic of the paradigms, we have categorized them into working memory–based versus long-term memory–based paradigms. In *working memory–based paradigms*, the test phase follows immediately after encoding of behavioral information, typically on a trial-by-trial basis. These paradigms appear most suited to investigate STI effects during spontaneous encoding of behavioral information. However, STI effects on task performance in these paradigms may predominantly rely on currently activated behavior–trait associations, which limits interpretability with regard to actor-specificity of trait inferences. In *long-term memory–based paradigms*, behavior encoding and test phase are structurally and temporally separated. More specifically, participants are first presented with trait-implying behavioral information, typically about several actors, and then—after an interval of varying duration that may or may not include filler tasks—enter a separate test phase. In consequence, performance in these paradigms additionally relies on long-term memory effects. Furthermore, the memory tests in these paradigms typically use some actor information as retrieval cue (photo, name, etc.), which allows for the conclusion that observed STI effects are indeed inferences about the actors rather than mere activations of trait–behavior associations (e.g., Todorov & Uleman, 2003).

Comparing the two groups of paradigms in our meta-analysis allows for a better differentiation of the processes relevant to STI formation and/or STI expression. For example, if STIs are formed indeed spontaneously upon encoding behavioral information (e.g., Uleman, Hon, et al., 1996), average effects of considerable size should already be visible in the working memory–based paradigms. The comparison to the average effect size in long-term memory paradigms would be equally informative: For example, smaller effect sizes in long-term compared with working memory–based paradigms could be taken as an indication that spontaneously formed impressions fade over time (and thus may have little impact on enduring impression formation). Also, such a result would support the assumption that spontaneous inference effects reflect the mere activation of behavior–trait associations rather than inferences about actors (e.g., Todorov & Uleman, 2004; Uleman et al., 2012). Finding comparable effect sizes in both groups of paradigms, on the other hand, would support the assumption that STI effects indeed represent actor-specific inferences (Todorov & Uleman, 2002). Finally, observing larger effect sizes in long-term memory–based paradigms than in working memory–based paradigms may indicate that impression-based biases of retrieval processes play a further role in STI effects.

Furthermore, the different experimental paradigms also allow for the implementation of different experimental manipulations, varying task instructions, different characteristics of the stimulus material and procedures, and the type of control conditions applied to examine STIs. In addition, some paradigms allow researchers to choose among different dependent variables (DVs) for investigating STI effects. These further methodological aspects of STI research also warrant meta-analytical investigation because they may also have theoretically relevant implications.

### Generalizability of STI Effects

Besides the methodological variations in STI research, the current meta-analysis also allows inspection of the generalizability of STI effects across different sample characteristics. Besides exploring gender and age differences, we focus on two characteristics that appear relevant for theoretical conceptualizations about the psychological foundation of STI effects in reference to cultural influences.

#### Cultural differences in STI effects

It has been repeatedly argued that person perception is culturally grounded and affected by cross-cultural differences in person construal and behavior attribution processes (e.g., Markus & Kitayama, 1991; Nisbett et al., 2001). For example, cultures with a predominantly *independent* person construal emphasize each individual’s uniqueness and autonomy in behavioral decisions, whereas cultures with predominantly *interdependent* person construal emphasize the effects of the social and material context on persons and accentuate the individual’s social and situational connectedness (Triandis, 2018). Related research has documented that participants from interdependent cultures are more likely to attribute behaviors to situational contexts than to actor’s personal traits (e.g., Masuda & Kitayama, 2004; Miller, 1984; Miyamoto & Kitayama, 2002, 2018; Morris & Peng, 1994), but recent research could not replicate these differences (Carstensen et al., 2021). Similarly, previous research has documented more pronounced STI effects in participants with independent compared with interdependent cultural backgrounds (e.g., Lee et al., 2017; Na & Kitayama, 2011; Newman, 1991; Shimizu et al., 2017; Zárate et al., 2001).

We investigate cultural differences first by comparing STI effects in samples from countries that score relatively high in individualism and independence (i.e., European, North American) with samples from countries scoring relatively high in collectivism and interdependence (i.e., Asian). We expect larger STI effects in individualistic samples compared with collectivistic samples. We complement this analysis using the continuous country aggregates of the individualism–collectivism dimension provided by Hofstede (2001) as a continuous predictor of the STI effect size. Finally, we include the country aggregates of the other five cultural value dimensions discussed by Hofstede (i.e., power distance, masculinity vs. femininity, uncertainty avoidance, long-term vs. short-term normative orientation, and indulgence vs. restraint) into an exploratory moderator analysis.

## Method

We preregistered this meta-analysis with the Open Science Framework in line with the checklist provided by PRISMA-P (Moher et al., 2015), adapted to our nonclinical research focus. There, we also provided an addendum after the coding process, specifying our analysis strategy and addressing several adjustments to the preregistered procedure and provide all data and analyses codes (https://osf.io/d5x9p).

### Literature Search and Eligibility Criteria

We used the terms “spontaneous trait inference” OR “spontaneous trait inferences” without limiting the publication date, searching the PsycINFO, Web of Science, ProQuest, and EBSCO databases and Google Scholar. In addition, we sent out open calls for unpublished studies on July 14 and 16, 2019, via the open forum of the Society for Personality and Social Psychology and the Email service of the European Association of Social Psychology. Finally, we conducted a backward search based on the reference lists of all included publications and a forward search screening all records citing included publications.

**Figure 1.**
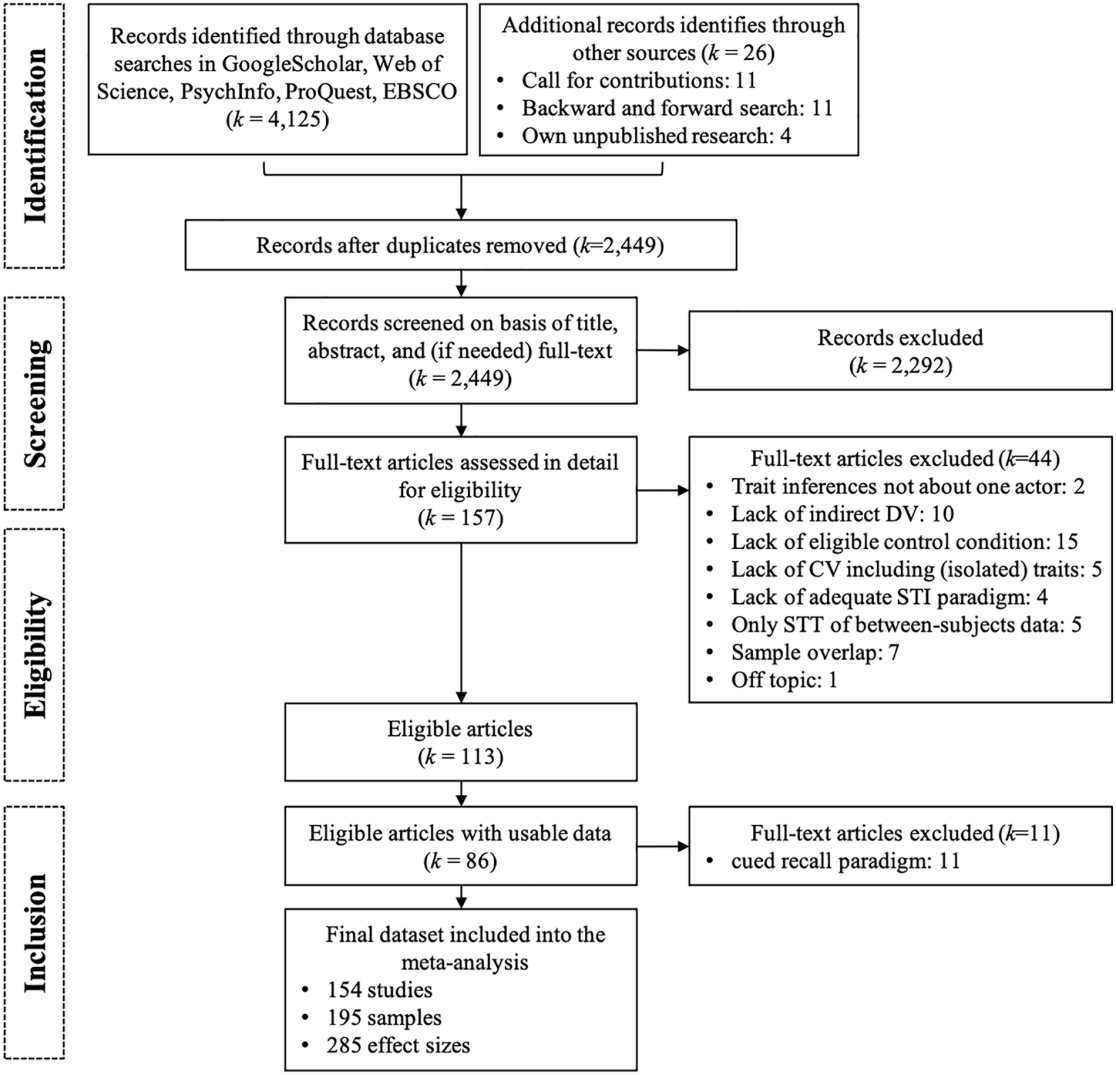
Flowchart of the coding process. *Note.* Exclusion reasons are specified on the study level. DV = dependent variable; STI = spontaneous trait inferences; STT = spontaneous trait transferences; ES = Effect sizes.

The database search was completed on October 25, 2018, and yielded 2,449 records after deleting duplicates (see Figure 1). Each record was screened for eligibility by two of the five independent coders, except for records in languages other than English, which were screened by only one coder each. Any discrepancies were resolved by discussion with the senior author (J.D.) if needed. We included studies meeting the following criteria:

1. Studies examining STIs from observed or reported human behavior. We excluded studies examining other inferences from behaviors (e.g., situation inferences, goal inferences, or other state inferences). We also excluded studies on trait inferences from information other than human behavior, for example, from faces, brands, contextual information, the behavior of animals, or attribute conditioning studies.
2. The traits had to be inferred about individual human actors. We excluded studies investigating trait inferences about groups of people, social categories, organizations, or animals.
3. The DV used had to refer to person-describing traits. We therefore excluded studies that used behavior predictions or person evaluations.
4. The measurement of STIs had to be indirect, for instance using error rates and/or response times in response to trait probes. We thus excluded studies using direct measures of trait impressions, such as open impression expressions, rating scales, or behavior predictions.
5. We excluded effect sizes based on brain imaging techniques (e.g., functional magnetic resonance imaging and electroencephalogram) given that effect sizes are difficult to compare with more frequently used DVs (at least in this meta-analytic context).
6. We included only studies conducted with nonclinical samples of native speakers.
7. In line with methodological criticisms (D’Agostino & Beegle, 1996) and incomparability of effect size estimation with all other paradigms, we excluded all studies that used the cued-recall paradigm (see detailed explanation in the Supplemental Material A). This exclusion criterion was not preregistered and applied only after a review of a previous version of this manuscript.

### Coding Procedure

We coded all eligible publications according to an adapted version of our preregistered procedure. Each study published in English or German was coded by two of the five first authors. Studies published in Portuguese were coded by the only Portuguese-speaking author (F.K.) of this article and verified by a second author (A.B.) with the help of a native speaker of Portuguese. We coded each eligible effect size separately, allowing for the inclusion of multiple contrasts (and other within-person conditions) within samples, multiple samples within studies, and multiple studies within publications. In cases of insufficient reporting of the required information, we contacted the authors of the respective publication.^1^

### Recorded Variables

Based on narrative reviews on STIs, we had initially identified several relevant moderator variables that could, however, not be coded from the literature. Furthermore, we excluded moderator levels from analyses, for which fewer than 5 effect sizes were available (see Supplemental Table S6 for a comprehensive list). In the following sections, we only describe those variables that were included in the reported analyses of this article. Our project in the Open Science Framework contains a comprehensive overview of the complete coding scheme along with the preregistration and addendum to the preregistration (see https://bit.ly/3cHAJa8 and the Supplemental Material B).

#### Experimental paradigms

We coded research paradigms as savings in relearning, false recognition, lexical decision, probe recognition, or word recognition. We also coded cued recall, which was, however, excluded from the main analyses.^2^ Because researchers sometimes used different labels for these paradigms, we based coding on the provided procedural descriptions (see Supplemental Material A). We additionally clustered the savings in relearning and false recognition paradigms as long-term memory–based because they implement structural and temporal separation between learning of actor-behavior information and memory test using various retention intervals with or without filler and distractor tasks. In this line, we clustered the probe recognition paradigm and the word recognition paradigm as short-term memory–based because they implement recognition tests immediately following each actor-behavior statement. The lexical decision paradigms were categorized as working memory–based if the lexical decision task was implemented on a trial-by-trial basis and as long-term memory–based when it was implemented as a block-wise testing procedure following an independent learning phase.

#### Instructions during behavior encoding

We coded the following instructions: impression formation, memorization, and familiarization. Note that several studies included manipulations of instructions but did not provide effect sizes separately for each condition such that we could not include them in this moderator analysis.

#### Contrasts

We coded all relevant STI contrasts for each given DV. We compared the following contrasts: implied versus implied other, implied versus novel trait, and implied versus antonym trait.

#### Dependent variables

We coded DVs as response times, error rates/accuracy, open recall of sentence parts, and recall of sentences. We were able to conduct moderator analyses including different DVs only for the probe recognition and the false recognition paradigm (RTs vs. error rates).

#### Stimulus and procedural characteristics

We recorded the following stimulus characteristics: number of tested traits (continuously), type of actor information (verbal and visual, verbal-only, and visual-only), type of verbal actor information (name, pronoun, and label/profession), type of verbal behavior description (sentence and paragraph), verbal behavior formulation (first person and third person). We coded the following procedural characteristics: filler task (absent, present), time interval between encoding and test phase continuously (in seconds for the working memory–based paradigms; in minutes for the long-term memory–based paradigms), and categorically (minutes vs. days in the savings in relearning paradigm).

#### Sample demographics

We recorded the mean age of the sample (continuously), sample type (children, students, adults), and the percentage of males within the sample (continuously).

#### Cultural differences

To establish the cultural background of the samples, we recorded the nationality of the sample and the sample ethnicity. If neither sample ethnicity nor nationality was explicitly reported, we used information about the location of the study or the first author’s affiliation as indirect indicators of sample nationality. We later categorized the apparent mono-cultural samples from Belgium, Canada, Germany, Italy, the Netherlands, Portugal, the United Kingdom, and the United States as “independent” and the monocultural samples from China and Japan as “interdependent.”^3^ Although we had additionally planned to include bicultural samples (e.g., Asian Americans, Asian Canadians), we had to exclude this subgroup analysis because only *k* = 4 effect sizes were eligible. Second, we retrieved the country average values of the six Hofstede (2001) dimensions from all included sample nationalities (https://www.hofstede-insights.com) as continuous indirect indicators of the samples’ cultural backgrounds.

#### Additional variables

Finally, we coded the publication status (published, unpublished), year of publication (continuously), and impact factor (continuously) of each record.

### Coding of Statistical Information

For each contrast, we coded the following statistical information, if available: means, standard deviations, standard errors, sample sizes, *t* value, *F* value, *p* value, correlation of measurements, Cohen’s *d*, η², η²_p_, degrees of freedom, and transformations applied to the data. Finally, we coded whether the results displayed an STI-conform effect.

### Effect Size Calculation

Given that almost all STI effects were based on within-subjects contrasts, we used the effect size *d*_z_ (Lakens, 2013), which represents the mean difference between two within-subject conditions standardized by the standard deviation of this difference score. We used the equations provided by Lakens (2013) and Rosenthal (1991) to calculate *d*_z_ from reported *t*-statistics and *F*-statistics:

(1) $$d_{z}=\frac{t}{\sqrt{n}}$$

(2) $$d_{z}=\frac{\sqrt{F}}{\sqrt{n}}$$

In cases where *t* or *F* values were not available, we calculated *t* values based on the available information (i.e., difference scores with standard deviations or standard errors, exact *p*-values, means with standard deviations, and the correlations between conditions). In cases where no exact sample size or degrees of freedom for the effect size of interest were available, the sample size was approximated by dividing the overall sample size by the number of groups (with random allocation using the statistics software R in case the number was not evenly divisible). The sampling variance of *d*_z_ was calculated with the Equations 3 and 4 provided by Gibbons et al. (1993):

(3) $$\sigma^{2}\left(d_{z}\right)=\frac{n-1}{\left(n-3\right)\cdot n}\cdot \left(1+d_{z}^{2}\cdot n\right)-d_{z}^{2}\cdot \frac{1}{C^{2}}$$

(4) $$C=1-\frac{3}{4\cdot \left(n-1\right)-1}$$

### Meta-Analytic Models

We included all relevant effect sizes from each sample such that many samples yielded multiple dependent effect sizes (e.g., when multiple DVs or STI contrasts were reported). This leads to statistical dependencies among effect sizes from the same sample. One way to address these statistical dependencies is the use of multilevel meta-analysis which models effect sizes as nested in samples (e.g., Cheung, 2014; Van den Noortgate et al., 2013). While this method does not model the dependencies exactly given the absence of full information about the covariance of effect sizes, simulation studies have generally supported its use (e.g., López-López et al., 2017; Moeyaert et al., 2017; Van den Noortgate et al., 2013). In addition to the inclusion of multiple effect sizes per sample, samples of participants were nested in studies, and studies were nested in publications. We therefore included “study” and “publication” as further levels in our multilevel meta-analysis (Fernández-Castilla et al., 2020). This resulted in a five-level meta-analytic model: Level 1: sampling variance of effect sizes; Level 2 (effect sizes): variation between effect sizes within samples; Level 3 (samples): variation between samples within studies; Level 4 (studies): variation between studies within publications; and Level 5 (publications): variation between publications. We implemented multilevel meta-analyses using the function rma.mv from the R package metafor (Version 2.0.0; Viechtbauer, 2010) of the open-source statistics software *R* (Version 3.4.3; R Core Team, 2018).

### Main Analysis

For the main analysis, we fitted the five-level meta-analysis without additional predictors. The intercept of this model quantified the average overall effect size of STIs. The heterogeneity of effect sizes was estimated by using an overall *Q*-test, interpreting the square root of the variance estimates from the five-level meta-analysis (i.e., standard deviations on each level: τ_2_, τ_3_, τ_4_, and τ_5_, respectively), and using likelihood ratio tests to assess fit improvement when individual variance parameters were included versus excluded. The variance on Level 3 (sample) was estimated as zero, and this parameter was removed from further analyses, yielding a four-level meta-analytic model.

### Moderator Analyses

All moderator analyses were implemented as four-level meta-analytic models with additional predictors. Given the high level of uncertainty with which variance components are estimated in the case of few effect sizes (Bender et al., 2018), moderator analyses based on few effects have to be interpreted with caution. In the first step, we examined the effects of paradigm (categorical; all levels with at least 5 effect sizes included) and paradigm type (long-term memory vs. working memory). Both were tested using likelihood ratio tests comparing the model with the predictor against a model without predictors. Given the pronounced effects of the paradigm, we controlled for it in all further moderator analyses. Thus, in line with the addendum to our preregistration (https://bit.ly/3cHAJa8), we created a base model with paradigm (categorical) as a predictor.^4^ Further moderators were tested using likelihood ratio tests comparing the base model with a model including the base predictor (paradigm) and the additional moderator.

For all moderators, we also examined regression coefficients but based the statistical inference on likelihood ratio tests. To facilitate interpretability, the most frequent level of the moderator was used as the intercept. Continuous moderators were *z*-standardized. In addition to regression coefficients, we also examined the empirical overall effect size for each moderator level (categorical predictors). These empirical effect sizes were computed as the intercept of a four-level model including only data in which the moderator of interest had the specified level.^5^ We provide 95% confidence intervals for all effect size estimates and regression coefficients.

In general, we applied the following rule for the inclusion of moderator levels: We included all moderator levels with five or more effect sizes and all continuous moderators with at least five effect sizes. Because the type of paradigm was included in the base model, we excluded all paradigms with less than five entries and all paradigms categorized as *other* for any given moderator analysis. For some individual moderator analyses, however, the approach of merely controlling for paradigm was not reasonable as the moderator could only be meaningfully examined within certain paradigms. In these cases, moderator analyses were implemented within individual paradigms or sets of paradigms. This was done for the following moderators: instruction (only meaningful for long-term memory paradigms), DV (examined within single paradigms), filler task (only meaningful for long-term memory paradigms), and the time interval between encoding and test phase (examined separately for working memory paradigms ranging in [milli-]seconds, long-term paradigms ranging in minutes, and for the savings in relearning paradigm ranging between minutes and days).

### Publication Bias

The potential role of publication bias (Rothstein et al., 2005) was examined using three approaches: First, we visually examined funnel plot asymmetry (using $1/\sqrt{n}$ rather than the standard error of the effect size as the y-axis of the funnel plots). Second, we included publication status (published in an academic journal vs. not) as a moderator while controlling for paradigm, similar to the other moderator analyses. A larger effect size for published studies would indicate the potential for publication bias. Third, we incorporated the meta-regression models PET (precision-effect test) and PEESE (precision-effect estimate with standard error; Stanley & Doucouliagos, 2014) to assess and correct for publication bias (for an overview see Carter et al., 2019) into our multilevel meta-analysis. PET includes the standard error of each effect size as a predictor, with the regression coefficient indicating potential publication bias and the intercept estimating the bias-corrected effect size. PEESE includes the variance rather than the standard error of the effect size as a predictor. To avoid biased testing (e.g., Gibbons et al., 1993; Pustejovsky & Rodgers, 2019), we used $1/\sqrt{n}$ and 1/n in place of the standard error (PET) and the variance (PEESE), respectively. We implemented PET and PEESE in the entire dataset while controlling for paradigm to examine whether there is evidence for small study effects overall. Moreover, we implemented PET and PEESE for each individual paradigm, examining both the evidence for small study effects and the corrected effect size. Especially for low numbers of studies, the latter has to be interpreted with caution given that these estimates can be relatively uncertain (Carter et al., 2019).

## Results

**Figure 2.**
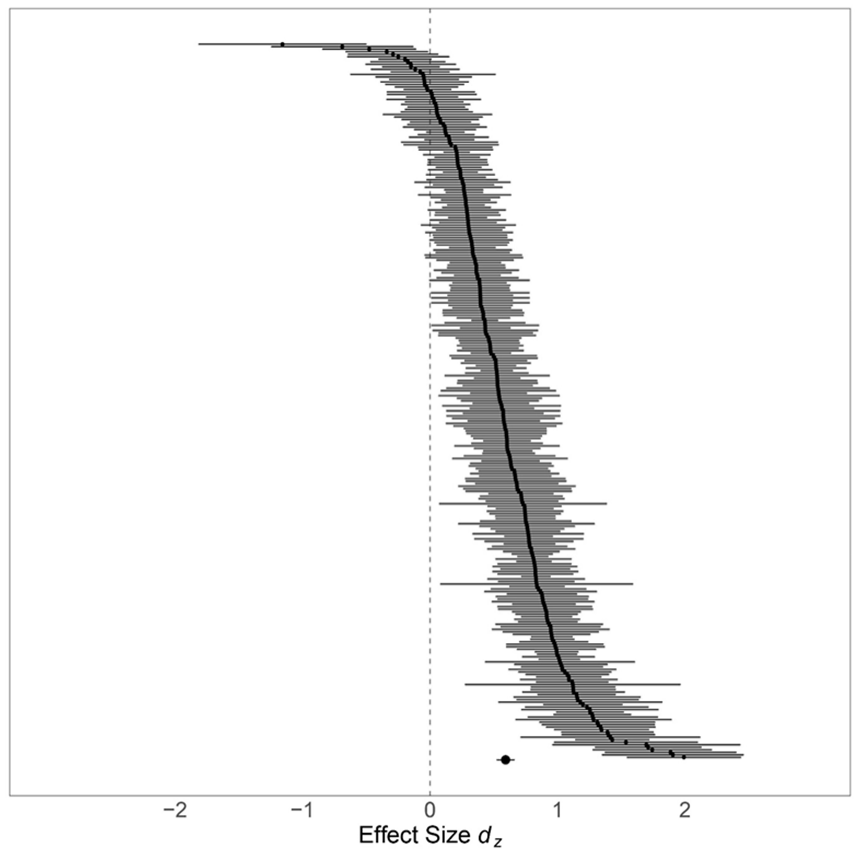
Forest plot of effect sizes included in the meta-analysis. *Note.* Shown are the individual effect sizes with 95% confidence intervals. Some of these effect sizes are dependent due to their nesting in sample, study, and paper. The overall effect size estimate corresponds to the estimate from our multi-level meta-analytic model.

The screening process resulted in 102 eligible publications (see Flowchart in Figure 1) of which 86 provided sufficient data for coding and effect size calculation for our main analyses (partly obtained via additional author contacts). We included 285 effect sizes from 195 independent samples in 154 studies with *N* = 13,630 participants. For the forest plot of all effect sizes, see Figure 2 and for a detailed overview of the included studies, see Table 2. The full reference list of all included studies is provided in Supplemental Material C. Additional analyses of data from eleven publications on studies using the cued recall paradigm are reported in the Supplement Table S1.

**Table 2.** Characteristics of All Studies Included in the Meta-Analysis.

Record	Studies	Samples	Contrasts	*N*	Aggregated ES	Paradigms	Country	Culture
Bassili & Smith (1986)	1	1	2	30	0.73	Word stem completion	Canada	Independent
Benrós (2018)	1	1	1	60	0.53	False recognition	Portugal	Independent
Boecking & Barnhofer (2014)	1	1	1	20	0.76	False recognition	UK	Independent
Brown & Bassili (2002)	1	1	1	97	0.74	Savings in relearning	Canada	Independent
Carlston and Skowronski (1994)	4	4	4	439	0.94	Savings in relearning	USA	Independent
Carlston et al. (1995)	3	3	3	215	1.08	Savings in relearning	USA	Independent
Casper et al. (2011)	3	3	4	142	0.35	Lexical decision (WM)	Germany	Independent
Costabile (2016)	1	3	3	89	0.73	Savings in relearning	US	Independent
Cowley (2005)	1	1	4	172	0.30	Other	Australia	Independent
Crawford et al. (2002)	3	6	6	425	0.89	Savings in relearning	USA	Independent
Crawford et al. (2007b)	1	1	2	47	0.61	Savings in relearning	UK	Independent
Crawford et al. (2007)	2	2	2	137	0.76	Savings in relearning	UK, USA	Independent
Crawford et al. (2008)	1	1	2	50	0.44	Savings in relearning	UK	Independent
Crawford et al. (2013)	2	5	10	239	0.72	Savings in relearning, False recognition	NA	Independent
D’Agostino & Hawk (1998)	1	1	1	12	1.12	Savings in relearning	USA	Independent
Elsbach et al. (2010)	1	1	1	60	0.78	False recognition	USA	Independent
Fiedler & Schenck (2001)	1	1	2	56	0.32	Word recognition	Germany	Independent
Fiedler et al. (2005)	3	3	5	140	0.34	Word recognition	Germany	Independent
Gill & Andreychik (2014)	1	1	1	49	0.31	Probe recognition	USA	Independent
Gonzalez et al. (2020)	4	4	11	271	0.61	False recognition	USA	Independent
Goren & Todorov (2009)	6	6	6	213	0.80	False recognition	USA	Independent
Ham & Vonk (2003)	2	2	2	103	1.18	Probe recognition, Other	Netherlands	Independent
Ham & Vonk (2011)	1	1	2	124	0.41	Other	Netherlands	Independent
Hamilton et al. (2015)	1	1	1	22	1.28	False recognition	USA	Independent
Johannes (2008)	2	2	4	113	0.55	Probe recognition	Germany	Independent
Kruse & Degner (2021)	4	4	6	800	0.52	False recognition, Probe recognition	Germany	Independent
Kruse (2017)	1	1	2	109	0.36	False recognition	Germany	Independent
Lass-Hennemann et al. (2011)	1	1	1	60	0.60	Savings in relearning	Germany	Independent
Lee & Uleman (2020)	1	2	4	126	0.79	Savings in relearning, False recognition	USA	Independent
Lee et al. (2017)	1	2	2	256	0.49	Savings in relearning	Canada, Japan	Independent, Interdependent
Levordashka and Utz (2017)	3	3	8	93	0.47	False recognition	Germany	Independent
Maass et al. (2001)	1	1	1	60	1.40	False recognition	Italy	Independent
Mangels & Degner (2022)	2	2	3	307	0.90	Probe recognition	USA, UK	Independent
Mangels, Stelter, & Degner (2022)	1	1	2	382	0.63	False recognition	Germany	Independent
McCall (2011)	1	1	1	61	0.67	False recognition	USA	Independent
McCarthy & Skowronski (2011)	2	5	5	307	1.31	False recognition	USA	Independent
McCarthy & Skowronski (2014)	4	4	4	543	0.73	False recognition	USA	Independent
McCarthy et al. (2013)	1	1	1	58	1.71	False recognition	USA	Independent
McCarthy et al. (2018)	2	2	2	278	0.64	Savings in relearning	USA	Independent
Na & Kitayama (2011)	1	2	4	100	0.59	Lexical decision (LTM)	USA	Independent, Interdependent
Nauts (2015)	3	3	3	418	0.70	Probe recognition	Netherlands, USA	Independent
Newman (1991)	1	2	2	70	0.02	Probe recognition	USA	Independent
Norman & Chen (2019)	1	1	2	112	0.13	Savings in relearning	USA	Independent
Nunes (2012)	1	1	1	62	0.99	False recognition	Portugal	Independent
Olcaysoy Okten & Moskowitz (2019)	2	2	4	147	0.23	False recognition	USA	Independent
Olcaysoy Okten & Moskowitz (2020)	1	1	1	80	1.12	False recognition	USA	Independent
Olcaysoy Okten (2015)	2	4	12	231	0.27	Probe recognition, False recognition	USA	Independent
Orghian (2017)	2	2	2	54	0.41	False recognition	UK	Independent
Orghian et al. (2017)	3	3	6	164	0.25	Modified free association paradigm	UK, Portugal	Independent
Orghian et al. (2018)	1	1	1	90	0.75	False recognition	Portugal	Independent
Orghian, de Almeida, et al. (2019)	2	2	2	192	0.47	False recognition	Portugal	Independent
Orghian, Ramos, et al. (2019)	3	3	7	225	0.37	Lexical decision (WM), Probe recognition, False recognition	Portugal	Independent
Otten and Moskowitz (2000)	1	1	2	14	0.34	Probe recognition	USA	Independent
Ramos (2009)	1	1	4	25	0.61	Probe recognition	USA	Independent
Ramos et al. (2018)	1	1	1	47	0.30	False recognition	USA	Independent
Rim et al. (2009)	3	3	3	170	1.17	False recognition	USA	Independent
Rim et al. (2013)	2	2	2	208	0.96	False recognition	USA	Independent
Risavy et al. (2010)	1	1	1	97	0.42	Lexical decision (WM)	Canada	Independent
Saribay et al. (2012)	2	2	3	86	0.79	Lexical decision (WM), False recognition	USA	other
Schneid et al. (2015)	1	1	1	85	0.92	Savings in relearning	USA	Independent
Shimizu & Uleman (2021)	2	6	6	398	0.72	False recognition	USA, Japan	Independent, Interdependent
Shimizu (2012)	2	6	9	386	0.37	Savings in relearning	Japan	Interdependent
Shimizu (2017)	2	2	2	272	0.45	Savings in relearning	Japan	Interdependent
Shimizu et al. (2017)	1	2	2	122	1.04	False recognition	USA, Japan	Independent, Interdependent
Todd et al. (2011)	2	2	3	126	0.23	Lexical decision (WM), Probe recognition	USA, Netherlands	Independent
Todorov and Uleman (2002)	6	6	10	308	0.71	False recognition	USA	Independent
Todorov and Uleman (2003)	3	3	3	86	1.42	False recognition	USA	Independent
Uleman, Hon, et al. (1996)	2	2	2	132	0.28	Probe recognition	USA	Independent
Varnum et al. (2012)	1	1	2	42	0.05	Lexical decision (LTM)	USA	Independent
Wang et al. (2015)	3	4	5	118	0.22	Probe recognition	China	Interdependent
Wang et al. (2016)	2	3	3	104	0.98	False recognition	China	Interdependent
Wang et al. (2017)	4	5	10	196	0.38	Probe recognition	China	Interdependent
Wang et al. (2018)	1	6	6	271	0.87	False recognition	China	Interdependent
Wells et al. (2011)	2	2	2	402	0.53	Savings in relearning	USA	Independent
Wentura & Greve (2005)	1	1	2	80	0.25	Lexical decision (WM)	Germany	Independent
Whitney et al. (1992)	1	1	1	48	1.06	Word stem completion	USA	Independent
Wigboldus et al. (2003)	1	1	4	45	0.54	Probe recognition	Netherlands	Independent
Wigboldus et al. (2004)	1	2	6	68	-0.14	Probe recognition	Netherlands	Independent
Wilkowski & Robinson (2010)	1	1	1	70	0.22	False recognition	USA	Independent
Wilkowski (2020)	1	1	1	93	0.56	False recognition	USA	Independent
Yang & Wang (2016)	2	2	4	130	0.28	Probe recognition	China	Interdependent
Zárate et al. (2001)	1	1	1	123	0.22	Lexical decision (WM)	USA	other
Zengel et al. (2017)	1	1	1	139	0.77	Savings in relearning	USA	Independent
Zhang & Fang (2016)	1	3	3	144	0.42	Probe recognition	China	Interdependent
Zhang & Wang (2013)	1	5	5	205	0.73	Probe recognition	China	Interdependent
Zhang & Wang (2018)	1	1	4	80	0.52	False recognition	China	Interdependent

*Note.* All information about sample size, effect size, paradigms, country, and cultural background were collapsed across studies, contrasts, and samples. Bicultural samples were excluded from the analysis with culture as moderator of the STI effect. ES = average effect size within each publication, aggregated across studies within a publication (within-subjects *d_z_*); *N* = total sample size within a publication; WM = working memory; LTM = long-term memory; NA = not available; STI = spontaneous trait inference. Full references are listed in Supplemental Material C.

### Main Analysis

The main analysis indicated an overall STI effect of *d*_z_ = 0.59, *Z* = 17.35, *p* < .001, with strong evidence for heterogeneity between effect sizes, *Q*(284) = 1,971.55, *p* < .001. The standard deviation of effect sizes was highest for effect sizes within samples, followed by variation between publications and between studies in publications (see Table 3). The corresponding standard deviations were substantial in magnitude, suggesting large amounts of variance that could be explained by moderators. The heterogeneity was estimated as zero on the level of samples within studies and was thus removed for further analyses.

**Table 3.** Results of the Main Analysis.

Component	*k*	Estimate [95% CI]	Test
*d_z_*		0.59 [0.53, 0.66]	*Z* = 17.35, *p* < .001
τ_5_	86	0.23	χ^2^(1) = 15.55, p < .001
τ_4_	154	0.13	χ^2^(1) = 2.15, p = .143
τ_3_	195	0.00	χ^2^(1) = 0.00, p = 1.000
τ_2_	285	0.25	χ^2^(1) = 165.38, p < .001

*Note.* τ_2_ = variation between effect sizes within samples; τ_3_ = variation between samples within studies; τ_4_ = variation between studies within publications; τ_5_ = variation between publications. CI = confidence interval.

### Moderator Analyses

We provide a comprehensive overview of descriptive statistics of all moderator variables along with intercorrelations of continuous moderators as well as an overview of co-occurrences of categorical moderator variable levels in Supplemental Tables S4 and S5, respectively.

#### Experimental paradigm

The analysis of the paradigm as a moderator of the overall STI effect indicated significant and substantial differences between experimental paradigms (see Table 4). In particular, the effect size was highest for the false recognition paradigm, followed by the savings in relearning paradigm. In general, long-term memory-based paradigms yielded significantly larger effect sizes (*d*_z_ = 0.69) than working memory-based paradigms (*d*_z_ = 0.38), χ^2^(1) = 19.63, *p* < .001. Nevertheless, STI effects were evident for all paradigms, ranging from *d*_z_ = 0.29 for lexical decision (WM) to *d*_z_ = 0.75 for false recognition, and significant in all cases except lexical decision (LTM), which may be attributable to the small number of studies (see Table 4).

**Table 4.** Experimental Paradigms as Moderator of STI effects.

Paradigm	Records	Studies	Samples	Contrasts	ES [95% CI]	β [95% CI]	Test
Long-term memory–based paradigms							
False recognition	38	69	86	120	0.75 [0.65, 0.86]		χ^2^(5) = 27.32, *p* < .001
Savings in relearning	20	32	44	54	0.62 [0.50, 0.74]	−0.15 [−0.29, 0.00]	
Lexical decision (LTM)	2	2	3	6	0.31 [−0.03, 0.66]	−0.39 [−0.78, –0.01]	
Working memory–based paradigms							
Probe recognition	20	31	41	69	0.39 [0.27, 0.52]	−0.32 [−0.46, –0.18]	
Word recognition	2	4	4	7	0.34 [0.18, 0.49]	−0.41 [−0.78, –0.05]	
Lexical decision (WM)	7	9	9	13	0.29 [0.21, 0.38]	−0.42 [−0.65, –0.19]	
Paradigm cluster							
Long-term memory	58	102	133	180	0.69 [0.61, 0.77]		χ^2^(1) = 19.63, *p* < .001
Working memory	27	44	54	89	0.38 [0.29, 0.47]	−0.29 [−0.41, –0.17]	

*Note.* ES = empirical effect size in a model including only effect sizes for which the moderator has the specified level, and no further predictor is included; β = difference in effect sizes in the multi-level model. If the two diverge, the model-based β should be assigned more weight. Note that for some records, data on multiple paradigms was available. CI = confidence interval; LTM = long-term memory; WM = working memory.

#### Instructions during behavior encoding

Only the following instructions could be used for moderator analyses: familiarization, memorization, and impression formation. As there was little variation in the instructions for working memory paradigms, the analysis of instruction was restricted to long-term memory paradigms.^6^ The overall moderation effect was significant, χ^2^(2) = 9.64, *p* = .008, with impression formation and familiarization yielding larger effect sizes (*d*_z_ = 1.16 and *d*_z_ = 0.72, respectively) than memory formation (*d*_z_ = 0.65). Note that the analysis for impression formation is severely limited by the small number of effect sizes (*k* = 5, see Table 5).

**Table 5.** Summary of Categorical Moderator Analyses.

Moderator	Moderator level	Paradigm	Records	Studies	Samples	Contrasts	ES [95% CI]	β [95% CI]	Test
Culture	Independent	False recognition, Savings in relearning, Probe recognition, Lexical decision (WM), Word recognition	67	121	137	200	0.62 [0.54, 0.70]		χ^2^(1) = 1.39, *p* = .239
	Interdependent	Probe recognition, False recognition, Savings in relearning	13	23	41	55	0.51 [0.39, 0.64]	−0.09 [−0.24, 0.06]	
Instruction	Memorization	False recognition, Lexical decision (LTM), Savings in relearning	32	52	62	90	0.65 [0.55, 0.75]		χ^2^(2) = 9.64, *p* = .008
	Familiarization	Savings in relearning, False recognition	20	31	47	62	0.72 [0.58, 0.85]	0.22 [0.02, 0.41]	
	Impression formation	Lexical decision (LTM), Savings in relearning, False recognition	4	4	4	5	1.16 [0.65, 1.66]	0.53 [0.16, 0.91]	
Contrast	Implied vs. implied other	False recognition, Probe recognition, Savings in relearning, Lexical decision (WM)	47	87	112	148	0.60 [0.51, 0.68]		χ^2^(2) = 9.65, *p* = .008
	Implied vs. novel trait	Savings in relearning, False recognition, Probe recognition, Word recognition, Lexical decision (LTM), Lexical decision (WM)	32	50	62	87	0.58 [0.48, 0.67]	0.05 [−0.07, 0.18]	
	Implied vs. antonym	Probe recognition, False recognition, Lexical decision (LTM)	4	4	5	10	0.22 [−0.09, 0.54]	−0.41 [−0.70, –0.13]	
DV (False recognition paradigm)	Error rates	False recognition	38	69	86	109	0.79 [0.69, 0.89]		χ^2^(1) = 38.08, *p* < .001
	RT	False recognition	5	7	9	11	0.02 [−0.37, 0.40]	−0.64 [−0.83, –0.45]	
DV (Probe recognition paradigm)	RT	Probe recognition	19	29	35	50	0.39 [0.25, 0.52]		χ^2^(1) = 0.02, *p* = .901
	Error Rates	Probe recognition	8	11	15	19	0.44 [0.24, 0.65]	0.01 [−0.15, 0.17]	
Sample Type	(mostly) Students	False recognition, Probe recognition, Savings in relearning, Lexical decision (WM), Word recognition	66	123	145	213	0.58 [0.51, 0.66]		χ^2^(2) = 0.43, *p* = .807
	Adults	False recognition, Probe recognition	11	15	17	26	0.69 [0.45, 0.93]	−0.06 [−0.24, 0.12]	
	Children	Probe recognition, False recognition, Savings in relearning	5	6	19	21	0.54 [0.34, 0.75]	0.02 [−0.20, 0.23]	
Type of actor information	Verbal, visual	False recognition, Savings in relearning, Probe recognition, Lexical decision (LTM), Lexical decision (WM), Word recognition	55	103	135	191	0.65 [0.57, 0.73]		χ^2^(2) = 0.19, *p* = .910
	Verbal	Probe recognition, Lexical decision (WM), False recognition	28	37	46	68	0.44 [0.33, 0.55]	0.03 [−0.17, 0.23]	
	Visual	Word recognition, False recognition	5	6	6	10	0.47 [0.29, 0.65]	−0.05 [−0.39, 0.28]	
Type of actor information: verbal	Pronoun	Savings in relearning, False recognition, Probe recognition, Lexical decision (WM)	37	63	85	111	0.67 [0.57, 0.77]		χ^2^(2) = 5.26, *p* = .072
	Name	False recognition, Probe recognition, Lexical decision (WM)	21	46	54	77	0.55 [0.42, 0.67]	−0.10 [−0.24, 0.05]	
	Label/profession	Probe recognition, False recognition	7	9	9	19	0.62 [0.45, 0.80]	0.17 [−0.08, 0.42]	
Type of behavior description: verbal	Sentence	False recognition, Probe recognition, Lexical decision (WM), Savings in relearning, Lexical decision (LTM)	62	109	139	199	0.58 [0.50, 0.67]		χ^2^(1) = 1.48, *p* = .224
	Paragraph	Savings in relearning, Probe recognition, False recognition	17	28	37	51	0.62 [0.49, 0.74]	0.12 [−0.07, 0.31]	
Verbal behavior description: wording	Third person	False recognition, Probe recognition, Lexical decision (WM), Savings in relearning	48	89	114	170	0.58 [0.48, 0.67]		χ^2^(1) = 0.11, *p* = .736
	First person	Savings in relearning, False recognition	24	43	56	73	0.68 [0.56, 0.79]	0.03 [−0.14, 0.20]	
Time interval between encoding and retrieval	Filler task	Savings in relearning, False recognition	30	49	71	86	0.73 [0.62, 0.83]		χ^2^(1) = 3.64, *p* = .057
	Test phase followed immediately after encoding	False recognition, Lexical decision (LTM)	18	37	46	65	0.66 [0.50, 0.81]	−0.21 [−0.43, 0.00]	
Time interval: unit (Savings in relearning)	Minutes	Savings in relearning	9	12	19	24	0.50 [0.35, 0.65]		χ^2^(1) = 1.83, *p* = .176
	Days	Savings in relearning	3	6	6	6	0.85 [0.71, 0.98]	0.37 [0.05, 0.69]	
Publication status	Published in an academic journal	False recognition, Savings in relearning, Probe recognition, Lexical decision (WM), Word recognition, Lexical decision (LTM)	65	121	159	215	0.60 [0.51, 0.68]		χ^2^(1) = 0.32, *p* = .571
	Not published in an academic journal	False recognition, Probe recognition, Savings in relearning	16	25	28	54	0.59 [0.47, 0.71]	−0.04 [−0.20, 0.11]	

*Note.* ES = empirical effect size in a model including only effect sizes for which the moderator has the specified level and no further predictor is included. β = difference in effect sizes in the multi-level model. If the two diverge, the model-based β should be assigned more weight. Paradigm = paradigms in which the moderator level occurs, ordered by frequency. All moderator and paradigm levels had to occur 5 times or more to be included. CI = confidence interval; WM = working memory; LTM = long-term memory; DV = dependent variable; RT = response times; .

#### Contrasts

There was a significant effect of contrast, χ^2^(2) = 9.65, *p* = .008. This was largely attributable to a much smaller effect size in studies using the implied versus antonym contrast, β = −0.41. There was no significant difference between the implied versus implied other and the implied versus novel trait contrast (see Table 5).

#### Dependent variables

Moderation by type of DV was analyzed separately within two paradigms given the intrinsic link between DV and paradigm: false recognition and probe recognition. For the false recognition paradigm, we observed robust STI effects with the error rate as the DV (*d*_z_ = 0.79) and essentially null effects for response times, χ^2^(1) = 38.08, *p* < .001 (see Table 5). In the probe recognition paradigm, however, effect size estimates for error rates and response times were nearly identical, χ^2^(1) = 0.02, *p* = .901 (see Table 5).

#### Stimulus and procedural characteristics

We further examined the following moderators: number of traits tested, actor information (both verbal and visual vs. verbal only vs. visual only), verbal actor information (pronoun vs. name vs. label/profession), verbal behavior description (sentence vs. paragraph), and verbal behavior wording (first person vs. third person). None of these moderators significantly improved model fit. Finally, neither the time interval between encoding and retrieval nor the presence or absence of a filler task was significantly related to the STI effect size (see Tables 5 and 6).

**Table 6.** Summary of Continuous Moderator Analyses.

Moderator	Observed range	Paradigm	Records	Studies	Samples	Contrasts	β [95% CI]	Test
Power distance^a^	35–80	False recognition, Probe recognition, Savings in relearning, Lexical decision (WM), Word recognition	76	138	173	245	−0.01 [−0.08, 0.05]	χ^2^(1) = 0.16, *p* = .687
Individualism versus collectivism^a^	20–91	False recognition, Probe recognition, Savings in relearning, Lexical decision (WM), Word recognition	76	138	173	245	0.05 [−0.01, 0.12]	χ^2^(1) = 2.47, *p* = .116
Masculinity versus femininity^a^	14–95	False recognition, Probe recognition, Savings in relearning, Lexical decision (WM), Word recognition	76	138	173	245	0.00 [−0.06, 0.07]	χ^2^(1) = 0.02, *p* = .888
Uncertainty avoidance^a^	30–99	False recognition, Probe recognition, Savings in relearning, Lexical decision (WM), Word recognition	76	138	173	245	−0.06 [−0.12, 0.00]	χ^2^(1) = 3.88, *p* = .049
Long-term versus short-term normative orientation^a^	26–88	False recognition, Probe recognition, Savings in relearning, Lexical decision (WM), Word recognition	76	138	173	245	−0.07 [−0.13, –0.01]	χ²(1) = 4.44, *p* = .035
Indulgence versus restraint^a^	24–69	False recognition, Probe recognition, Savings in relearning, Lexical decision (WM), Word recognition	76	138	173	245	0.04 [−0.02, 0.11]	χ^2^(1) = 1.81, *p* = .178
Age (in years)	8.5–44.2	False recognition, Probe recognition, Savings in relearning, Lexical decision (WM)	43	67	91	133	−0.01 [−0.08, 0.07]	χ^2^(1) = 0.04, *p* = .851
Gender (% male participants)	8.3–70.6	False recognition, Probe recognition, Savings in relearning, Lexical decision (WM)	49	79	103	158	−0.02 [−0.08, 0.05]	χ^2^(1) = 0.28, *p* = .596
Number of traits tested	2–80	False recognition, Probe recognition, Savings in relearning, Lexical decision (WM), Word recognition, Lexical decision (LTM)	80	145	186	268	0.02 [−0.03, 0.07]	χ^2^(1) = 0.57, *p* = .452
Time interval: WM paradigms (in s)	0.05–1	Probe recognition, Lexical decision (WM)	18	27	34	52	0.04 [−0.07, 0.15]	χ²(1) = 0.45, *p* = .501
Time interval: LTM paradigms (in min)	2–27.2	False recognition, Savings in relearning	21	29	46	56	0.11 [0.00, 0.22]	χ^2^(1) = 3.31, *p* = .069
Publication year	1991–2021	False recognition, Probe recognition, Savings in relearning, Lexical decision (WM), Word recognition, Lexical decision (LTM)	75	136	176	247	−0.09 [−0.15, –0.03]	χ^2^(1) = 8.33, *p* = .004
Impact factor	0.676–6.335	False recognition, Savings in relearning, Probe recognition, Lexical decision (WM), Word recognition, Lexical decision (LTM)	62	115	149	205	0.10 [0.03, 0.16]	χ^2^(1) = 7.72, *p* = .005

*Note.* β = linear effect of the *z*-standardized moderator in the multilevel model. Paradigm = paradigms in which the moderator occurs, ordered by frequency. Moderators with at least 5 effect sizes were included. CI = confidence interval; WM = working memory; LTM = long-term memory.

a Dimensions of national culture (Hofstede, 2001).

#### Sample demographics

Cultural background did not significantly moderate the overall STI effect, χ²(1) = 1.39, *p* = .239, with substantial and significant STI effects both for samples from independent cultures (*d*_z_ = 0.62) and for samples from interdependent cultures (*d*_z_ = 0.51; Table 5). Similarly, the Hofstede dimension individualism versus collectivism was not significantly related to the STI effect size, β = 0.05, χ² (1) = 2.47, *p* = .116. Thus, while STI effect sizes were descriptively slightly smaller for participants in collectivistic than individualistic cultures, these differences were not significant.^7^

Furthermore, two of the other dimensions of cultural value variations proposed by Hofstede were significantly associated with the STI effect size, long-term orientation, and uncertainty avoidance, which indicated that STI effects were larger in countries that tend toward low values on these dimensions (see Table 6).

The sample characteristics such as mean age, percentage of male participants, and sample type (children vs. students vs. adults) were all unrelated to the mean STI effect size (*p*-values ≥ .596, see Table 5 and Table 6). Substantial and significant STI effect sizes were observed in all age groups.

### Publication Bia

**Figure 3.**
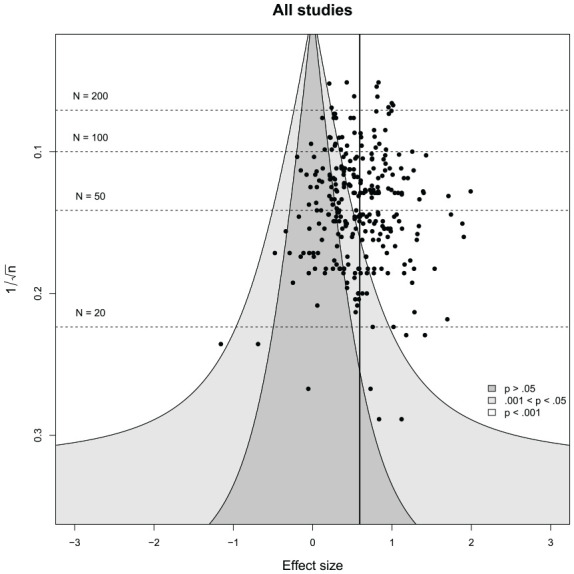
Funnel plot depicting all effect sizes included in the meta-analysis. *Note.* Depicted is a funnel plot including data from all experimental paradigms.

**Figure 4.**
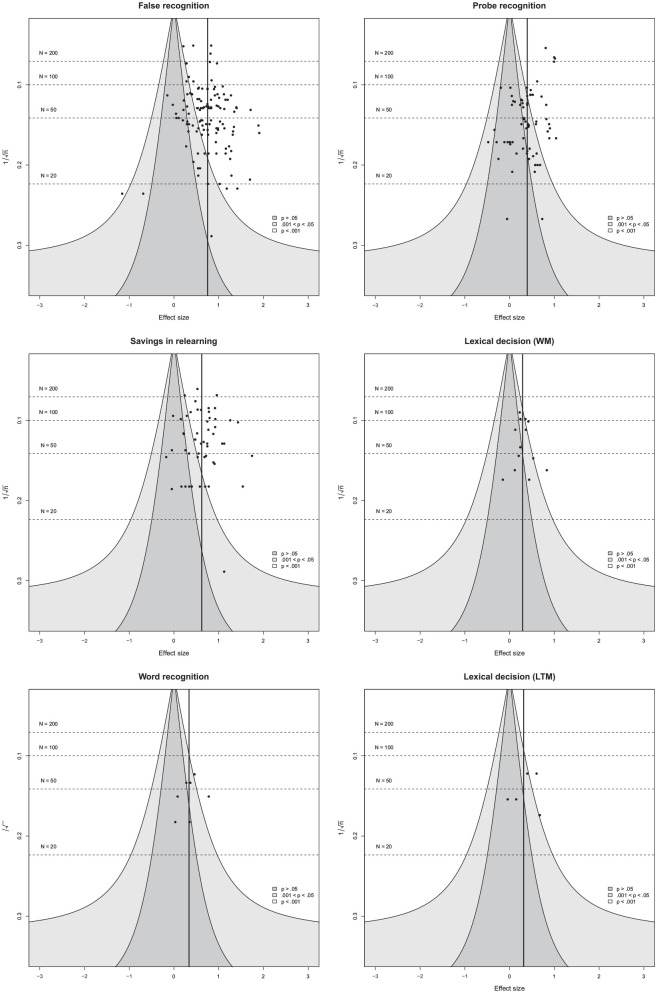
Funnel plots for each experimental paradigm. *Note.* Depicted are funnel plots for the different experimental paradigms. Two effect sizes from LTM-based lexical decision paradigms are numerically very similar and hence overlapping. WM = working memory; LTM = long-term memory.

A visual inspection of the funnel plots (see Figures 3 and 4) indicated no obvious funnel plot asymmetry. Similarly, publication status (published vs. unpublished) was unrelated to the overall STI effect size, χ²(1) = 0.32, *p* = .571. Average effect sizes for published and unpublished studies were almost identical (see Table 5). Year of publication and journal impact factor were significantly associated with the overall effect size such that earlier reports and reports published in higher impact journals yielded somewhat higher effect sizes (see Table 6). Adding quadratic effects did not result in significant fit improvement (see Supplemental Table S3), although the quadratic effect of year was descriptively negative (Supplemental Figures S1 and S2). Finally, the PET and PEESE analyses supported the robustness of our results to publication bias. Neither 1/n (PEESE) nor $1/\sqrt{n}$ (PET) were significant effect size predictors. For paradigm-specific analyses, neither predictor was significantly related to the effect size, again indicating no evidence for bias (see Table 7). Corrected effect sizes were substantial in size except for lexical decision (LTM; PET and PEESE), which was estimated with very high uncertainty given the low number of studies (see Table 7). For PET, corrected effect sizes remained significant for three paradigms, and for PEESE—which yields better estimates if the true overall effect size is nonzero—corrected effect sizes remained significant in all cases except for lexical decision (LTM). Overall, our analyses therefore yield no evidence of small study effects and indicate the robustness of STI effects to publication bias.

**Table 7.** Analyses of Small Study Effects.

Type	Paradigm level	Records	Studies	Samples	ES	ES_adjusted_ [95% CI]	β [95% CI]
$\frac{1}{n}$ : PEESE	Overall	81	146	187	269		−0.54 [−4.75, 3.67]
	False recognition	38	69	86	120	0.69 [0.53, 0.85]	3.24 [−3.04, 9.52]
	Probe recognition	20	31	41	69	0.46 [0.26, 0.67]	−3.25 [−10.92, 4.41]
	Savings in relearning	20	32	44	54	0.63 [0.43, 0.83]	−0.51 [−10.06, 9.05]
	Lexical decision (WM)	7	9	9	13	0.30 [0.10, 0.50]	−0.31 [−11.92, 11.31]
	Word recognition	2	4	4	7	0.59 [0.09, 1.10]	−11.63 [−33.65, 10.39]
	Lexical decision (LTM)	2	2	3	6	0.10 [−0.49, 0.70]	9.60 [−11.90, 31.11]
$\frac{1}{\sqrt{n}}$ : PET	Overall	81	146	187	269		−0.17 [−1.42, 1.07]
	False recognition	38	69	86	120	0.57 [0.30, 0.83]	1.37 [−0.46, 3.20]
	Probe recognition	20	31	41	69	0.62 [0.28, 0.96]	−1.62 [−3.89, 0.65]
	Savings in relearning	20	32	44	54	0.66 [0.30, 1.02]	−0.31 [−3.06, 2.44]
	Lexical decision (WM)	7	9	9	13	0.29 [−0.09, 0.67]	0.00 [−3.05, 3.04]
	Word recognition	2	4	4	7	0.86 [−0.15, 1.87]	−3.56 [−10.37, 3.25]
	Lexical decision (LTM)	2	2	3	6	−0.09 [−1.09, 0.91]	2.77 [−3.59, 9.12]

*Note.* Shown is an analysis of small study effects. PEESE provides better estimates when the true effect size is nonzero, which is likely the case here. ES_adjusted_ = an estimate of the bias-corrected effect size, β = indicates the presence of small study effects (positive value = larger effects in smaller studies). CI = confidence interval; PEESE = precision-effect estimate with standard error; WM = working memory; LTM = long-term memory; PET = precision-effect test.

## Discussion

The overarching goal of this meta-analysis was to investigate the average effect size and the relative robustness of the STI effect and to identify potential moderators that may have both conceptual and methodological relevance. We first briefly discuss the results of the overall effect size analysis and then provide more detailed reflections on the implications of the results of the moderation analyses.

### Overall Effect Size of STIs

Our results demonstrate a significant average STI effect estimate of moderate to large size (*d_z_* = 0.59). Translated into a common language effect size, the observed results imply that approximately 72% of all participants would be expected to numerically show an STI effect. This observed effect is comparable to a moderate between-subjects effect size of approximately *d* = 0.59 (Lakens, 2013),^8^ which is substantially larger than the median effect size reported in social psychology (estimated as *d* = .36; Lovakov & Agadullina, 2021).

Taken together, our results support previous conclusions in narrative reviews that the STI effect is a robust phenomenon (e.g., Uleman et al., 2008). Furthermore, publication bias did not affect the overall effect size. Nevertheless, studies published earlier and in journals with higher impact factors reported somewhat larger effect sizes, thus indicating a decline effect known in many research fields (e.g., Schooler, 2011). Importantly, STI effects were similar for published and unpublished as well as for studies with small and large samples. In light of concerns regarding the replicability of published psychological effects (e.g., Open Science Collaboration, 2015), this finding provides further meaningful support for the robustness of STIs across the literature.

Our analyses further demonstrated significant heterogeneity across all effect sizes as well as between publications and between effect sizes within samples, indicating that the strength of the STI effect was influenced by several other factors. We focus our discussion first on methodological factors and their implications and then discuss sample characteristics.

### Experimental Paradigm

Our moderator analysis of classes of experimental paradigms revealed significant STI effects in both long-term memory–based and working memory–based paradigms that were, however, significantly larger in the long-term memory–based paradigms. These results first of all indicate that STIs are detectable both at the initial encoding of behavioral information and at the retrieval of person information. These results also indicate that STI effects are stable over time—or at least over the time periods typically investigated in the long-term memory–based paradigms (ranging from minutes to days).

Besides the generally robust STI effects across the different memory-based paradigm clusters, we also observed substantial effect sizes in all individually tested paradigms, nearly all of which were significant. In further support of robustness, STIs seem to be measurable under various methodical settings, even under conditions where task performance hinders STI formation. Observing STIs under performance-hindering conditions has been viewed as the most robust evidence for the unintentionality of these inferences, as there is no incentive to perform unsuccessfully in a task (see also Carlston & Skowronski, 1994; Todorov & Uleman, 2002). Interestingly, the false recognition paradigm and the probe recognition paradigm that both operationalize STI effects via impaired task performance both elicited the highest STI effects within their clusters. These findings further eliminate the alternative explanation that attributes STI effects to deliberate strategies and underline the assumed nonintentionality of STIs (Uleman, Newman, & Moskowitz, 1996).

The observed effect size difference between the two clusters of working memory–based and long-term memory–based experimental paradigms can be interpreted in several ways. First and most straightforward, one may speculate about the potentially additive and/or interacting contributions of encoding and retrieval processes to person inference effects. Previous research has excluded mere behavioral recall and retrieval-based person inferences during testing as alternative explanations of STI effects (e.g., Todorov & Uleman, 2002). Nevertheless, it is still possible that such processes augment STI effects—leading to larger effect sizes in the long-term memory–based paradigms as compared to the working memory–based paradigms. Second, there are several further procedural differences between long-term memory– and working memory–based paradigms that offer other compelling interpretations and point toward important process characteristics of the formation and retrieval of STIs. One of them is the stimulus representation with or without actor images and another may be the relative difficulty of task completion in the different paradigms.

With regard to actor images, the included long-term memory–based paradigms present actor images along with behavioral statements and later use these actor images as prompts for measuring STI effects (which does not apply to the excluded cued recall paradigm, see Supplemental Material A). In contrast, working memory–based paradigms rarely employ actor images. Using actor images may facilitate the formation of actor-trait links at encoding, for example, because it increases the salience of the actor, which in turn may lead to larger STI effects. The effect size differences between both groups of paradigms may therefore be interpreted as further support that STI effects are more than mere effects of behavior-trait categorization at encoding of behavioral information but indeed represent actor-trait links based on inferences about the actors (e.g., van Overwalle et al., 1999). Alternatively, the presentation of actor images during encoding might simply increase the general stimulus salience, resulting in an unspecific processing advantage that—as a side effect—also supports the formation of STIs (e.g., Uleman et al., 1993) but is not necessarily specific to STI formation. Note, however, that when we specifically investigated the presence versus absence of actor images as moderator variable, we did not find significant differences in STI effects. This questions whether this variable is responsible for the difference between working memory–based and long-term memory–based STI effect sizes.

A second methodical confound between both types of paradigms refers to relative task difficulty. While long-term memory–based paradigms typically present behavioral information about a number of actors that are presented block-wise before proceeding to the STI test phase, working memory paradigms implement behavioral encoding and trait-inference testing consecutively on a trial-by-trial basis. Hence, the number of items learned and tested simultaneously is greater in designs with block-wise encoding. Along with longer time intervals between encoding and testing and/or interruptions by filler tasks, this may cause long-term memory–based paradigms to be generally more difficult than working memory paradigms. This increased difficulty might circumvent the problem of ceiling effects caused by highly accurate and fast responding in the easier working memory–based tasks and therefore lead to larger effect sizes. The comparison of the probe recognition paradigm and the false recognition paradigm serves as a good example here. Procedurally, these paradigms are highly similar with the main difference being an increased task difficulty because of block-wise stimulus encoding and testing in the false recognition paradigm versus the trial-by-trial procedure in the probe recognition paradigm. In line with this notion, Todorov and Uleman (2002) showed false recognition rates of over 40% for implied traits in the false recognition paradigm, while false recognition rates in probe recognition paradigms are typically below 10% (e.g., Wang & Yang, 2017). With less potential for ceiling effects, this may explain why false recognition yields higher effect sizes than probe recognition. While conceivable, this interpretation was not supported by the moderator analyses that investigated length of time intervals and absence versus presence of filler tasks in the long-term memory–based paradigms, which one may use as indirect indicators of task difficulty. Neither variable significantly moderated the STI effect size. This potential factor should be addressed in future systematic empirical investigations, along with further potential systematic differences between groups of paradigms.

In conclusion, results from the paradigm moderator analyses indicate that STIs are formed immediately at encoding and subsequently stored in long-term memory. Our speculations about the observed effect size differences between working memory–based and long-term memory–based experimental paradigms illustrate that a number of procedural aspects might play a pivotal role in explaining these differences. As these procedural choices are highly intertwined with other characteristics of the experimental paradigms, future research should more systematically test these variations to identify task characteristics that are responsible for effect size differences and their implications for conclusions regarding the underlying mechanisms of STIs.

### Instructions During Behavior Encoding

We had additionally analyzed variations of instructions as potential moderators of STI effects. The most frequent variations were the instruction to memorize or familiarize with the actor and behavior information (e.g., Carlston & Skowronski, 1994; Goren & Todorov, 2009), which are typically compared with explicit instructions to form an impression of the actor (e.g., Uleman, 1987). The major rationale behind these variations was that finding significant STI effects in the familiarization and memorization conditions supports the assumption of *spontaneous* impression formation because participants have no explicit goal to form any impression of the actors. It was furthermore argued that instructions explicitly triggering intentional and deliberate impression formation may augment STI effects (Uleman, 1987) and thus represent the upper threshold for the STI effect size.

Contrasting previous findings (e.g., Carlston et al., 1995; Carlston & Skowronski, 1994; Ramos et al., 2018; Todorov & Uleman, 2002; Todorov & Uleman, 2004), our moderation analysis demonstrated that STI effect sizes were significantly affected the type of instruction during encoding. Specifically, effect sizes were large when participants received the instruction to actively form impressions about actors as compared with moderate effect sizes when participants were instructed to merely familiarize or memorize behavioral information. This result somewhat supports Uleman’s (1987) assumption that impression formation instructions evoke stronger trait inferences. Note, however, that this analysis was based on only few effect sizes (i.e., *k* = 5 for impression formation) because several studies using multiple instructions did not provide sufficiently detailed test statistics for individual conditions to allow inclusion into this moderation analysis. While this question itself may spark little interest for future research, we deem it important to more systematically investigate the comparative influence that spontaneous nonintentional trait inferences and deliberate intentional trait inferences have on overall impression formation in person perception or even their downstream effects on interpersonal interaction dynamics.

It should also be noted that several further theoretically important variations of instructions could not be investigated in our meta-analysis because too few effect sizes were available. Specifically, instructions to focus on the situation, to actively suppress dispositional inferences as well as cognitive load manipulations could not be included. Especially the latter manipulation has yielded controversial results as early work on the automaticity of STIs found no effect of cognitive load (e.g., Winter et al., 1985) while other investigations found evidence for an influence of cognitive load on STI formation (e.g., Wells et al., 2011).

### Contrasts

As a further methodologically relevant variable, we investigated whether the chosen control condition and thus type of contrast significantly moderated the STI effect size. We indeed observed a significant moderation, which can be largely attributed to a smaller effect size in the implied versus antonym contrast while the implied versus implied other and implied versus novel trait contrasts did not significantly differ. These findings contradict previous assumptions that contrasts with antonyms should yield larger effect sizes because the trait inference might also imply the actor lacking the opposite of the implied trait (i.e., the antonym trait, Todorov & Uleman, 2002). Our analysis, however, is only based on *k* = 10 effect sizes of which *k* = 6 are from a single publication investigating the role of antonyms but also of stereotypes which may have decreased the effect sizes (Wigboldus et al., 2004).

Somewhat unexpected, our analyses yielded no difference between contrasts using traits implied by behavioral statements within the same task (implied-other) versus novel traits unrelated to all presented behavioral statements. Comparing implied traits with implied-other traits as control condition has been frequently advocated as gold standard because it controls for the unspecific familiarity or accessibility that trait words may acquire during the encoding phase. Using novel traits as control contrast, on the contrary, has been argued to artificially increase effect size estimations for STIs due to their relatively lower familiarity and/or accessibility. Our results, however, indicate that novel traits did not result in larger effect size estimates than implied-other traits. Thus, STI effect size estimation does not appear to be influenced by the relative familiarity or accessibility of the traits used in the control condition. Potential differences between the two contrasts, if they exist, are thus likely small, which provides higher levels of flexibility for researchers in choosing fitting control conditions within the constraints of their research questions.

### Dependent Variable

As the choice of DV to measure STIs is highly contingent on the experimental paradigm, we only evaluated differences within two paradigms that provided sufficient variation: false recognition and probe recognition. Our results for probe recognition indicate that the choice of DV did not significantly alter the detectability of STIs. In this paradigm, STI effects of comparable size were observable for accuracy as well as response times. This suggests that response times and accuracy serve as reliable measures for STIs, and it should thus become standard practice to record, analyze, and report both.

In contrast, the choice of DV in the false recognition paradigm did make a significant difference, as significant STI effects were only observed in accuracy measures (i.e., error rates) and not in response times. Therefore, we recommend focusing on accuracy measures in this paradigm.

### Stimulus and Procedural Characteristics

Our moderation analyses encompassed a number of further methodological aspects. The number of traits tested, presenting the actor information verbally or visually (or in both ways), describing the actor with the use of pronouns, names, or labels, using sentences or paragraphs for the behavior description, and presenting the behavior description in first or third person did not significantly moderate the STI effect size. These commonly used methodological variations appear to have limited effects on the STI effect size, again emphasizing the robustness of STI effects. Nevertheless, our results cannot rule out that further variations that are less commonly used in the literature can affect STI effects, which may be systematically investigated in future research.

The STI effect was also related neither to whether a filler task was used between encoding and retrieval nor to the time interval between encoding and retrieval. This shows that STI formation is not only robust against methodological variations but also against distractions (filler tasks) and longer time intervals between encoding and retrieval (e.g., days in the savings in relearning paradigm). Numerically, effects were even slightly larger for longer time intervals and when filler tasks were used. Nevertheless, it should be noted that studies systematically testing for effects of filler tasks and length of time intervals while holding all other conditions constant might still find effects of these factors as, for example, Carlston and Skowronski (1994) found that the STI effect weakened when the interval between encoding and retrieval was 7 days.

### Sample Demographics

Neither age, gender, nor the sample type (e.g., student sample or children sample) significantly moderated the effect size of STIs. This is in line with work indicating that children already form STIs (e.g., Newman, 1991; Shimizu, 2012; Wang et al., 2018) and that STIs are present in nonstudent adult samples (e.g., McCarthy et al., 2013; Olcaysoy Okten, 2015). Although few studies already began to investigate the developmental course of STIs (e.g., Wang et al., 2018; Zhang & Fang, 2016) some demographic groups are underrepresented in our sample, especially young children (younger than 8 years) and elderly people. Nevertheless, the lack of age, gender, and sample type effects underlines that STI effects are not only robust across several methodological variations but also evident in different demographic groups.

### Cultural Differences Between samples

Contrary to expectations, participants’ cultural background was not a significant moderator of STIs. Although results descriptively point in the hypothesized direction, samples with an independent cultural background did not show a substantially larger STI effect size than samples with an interdependent cultural background. Similar results were obtained when employing country aggregates of the individualism versus collectivism dimension provided by Hofstede (2001) as continuous predictors. An effect of (in)dependence as cultural orientation was only observed when restricting analyses to the studies that conducted a direct sample comparison (see Supplemental Table S2), but their number is too low to draw reliable meta-analytic conclusions. This result is at odds with early theoretical conceptualizations of how cultural differences along the individualism versus collectivism dimension might influence the formation of STIs (e.g., Moskowitz, 2005; Uleman, Newman, & Moskowitz, 1996). Depending on differences in independent versus interdependent self- and person construal (e.g., Markus & Kitayama, 1991, 2010) and the related tendency to spontaneously use traits to explain observed behavior, it has been theorized that STIs should occur more frequently in samples with a predominantly individualistic (e.g., European and North American) cultural background compared with samples having a rather collectivistic (e.g., Asian) cultural background (e.g., Moskowitz, 2005; Uleman et al., 2008). The lack of significant cultural differences in the present meta-analysis is largely attributable to recent research conducted in Chinese (Lee et al., 2017; Wang et al., 2015, 2016, 2018; Wang & Yang, 2017; Yang & Wang, 2016; Zhang & Fang, 2016; Zhang & Wang, 2013, 2018) and Japanese samples (Shimizu, 2012; Shimizu & Uleman, 2021). Thus, the results of this meta-analysis suggest that STIs might not be as dependent on cultural differences in person construal as previously theorized. Such findings are in line with recent cross-cultural research that did not find the expected difference in person versus situational attribution between U.S. American and Chinese participants (Carstensen et al., 2021). Our analyses did, however, reveal a moderating effect of two other dimension of cultural value differences: long-term versus short-term normative orientation and low versus high uncertainty avoidance. High scores on normative orientation dimension indicate a tendency among people to endorse efforts in modern education to prepare for the future, whereas low scores indicate a tendency to prefer established traditions over societal change. Scores on the uncertainty avoidance dimension indicate the relative comfort or discomfort with uncertainty, ambiguity and the unpredictability of the future and the relative strictness, rigidness, or tolerance toward aberrations from codes of belief and behavioral codes (Hofstede, 2001). Given the exploratory character of this non-preregistered analysis and our large number of hypothesis tests, this result should be interpreted with caution.

In general, the moderator analyses on cultural differences included data from only nine countries. Conclusions about (the lack) of cultural differences thus have to be drawn with caution. Clearly, the theoretical assumption that cultural values and/or differences in person construal may affect spontaneous impression formation should be investigated more systematically in the future, not just by conducting mere cross-country comparisons that are open to many confounding variables but by inclusion of theoretically derived measures of cultural variations as mediator variables. Instead of relying on mere country aggregates of cultural values, this research should adopt a multilevel approach in which not only group aggregates of cultural values but also individual levels of conformity to these values are included into analyses.

### Limitations and Outlook

Multiple limitations need to be kept in mind in regard to our statistical moderator analyses. First, given insufficient codable effect sizes for several moderator levels, we had to exclude potentially relevant moderators from our analyses. This is partly attributable to suboptimal reporting and data availability standards that have only begun to improve in the last decade. Therefore, our results cannot provide a comprehensive view of the impact of different moderators on the STI effect size that we initially aimed for (see Supplemental Table S6 for a summary). For instance, we could not include cognitive load as an important instruction variation during behavior encoding. Furthermore, this meta-analysis did not include potential moderators regarding individual differences (e.g., perceivers’ expectations of observed behavior based on group membership and associated stereotypes) or item-based stimulus characteristics (e.g., valence or extremity of presented behavior). As a substantial amount of variance in the STI effect is still left unexplained, this stresses the need for highly powered studies systematically investigating relevant moderators in this research domain, along with improved reporting standards and open data accessibility.

Second, some of our moderator analyses suffer from limited statistical power due to an underrepresentation of datasets covering certain moderator variables or some of their levels, resulting in uncertain effect size estimates. In some cases, this can be attributed to an overall low occurrence of specific procedures in this field of research. However, we also identified several studies that investigated central moderator variables (e.g., instruction during behavior encoding) but that did not provide enough information for effect size calculations for single conditions—mostly when they did not observe significant differences between conditions. This supports the need for more transparent reporting standards, which are already being implemented more widely in psychology in recent years (e.g., Appelbaum et al., 2018).

Third, the correlational nature of meta-analytic moderator analyses leaves the possibility that the presented moderator effects are compromised by confounding variables, for example, differences in stimulus selection between samples or unreported sample characteristics. In contrast, however, our meta-analytic moderator analyses have the strength that they include far more effect sizes than is possible in individual reports. Nevertheless, large-scale experimental studies are needed to gauge our moderator findings, especially where unclear or unexpected (e.g., on the impact of cultural values or of instructions during encoding).

Next to the limitations of our analyses, some shortcomings of the STI literature itself need to be discussed. One main issue refers to the multitude of different stimulus sets that are employed at varying frequencies and lack systematic overall characterizations. As pointed out by Levordashka and Utz (2017), these stimulus sets seem to differ in both extremity and distinctiveness of the described behaviors which might result in STIs of varying strength and thus influence the robustness of the STI effect size. The variance and generalizability across stimuli are rarely investigated (but see Uleman, Hon, et al., 1996; see also the debates regarding the “the-language-as-a-fixed-effect fallacy,” as introduced by Clark, 1973). Ideally, raw data of individual studies and their full stimulus materials would be available for meta-analyses, which would allow systematically testing if and to what extent STI effects are affected by such stimulus characteristics. Because raw data were typically not available, we are left to state that despite several decades of highly active research, we are unable to draw firm conclusions with regard to many of these methodological issues. We hope that the increasing use of open science practices will enable such analyses in the future.

Future research on STIs should also consider ways of including stimuli as random variables into analyses to generalize results across stimuli as well (see Judd et al., 2012; Uleman, Hon, et al. 1996). This also implies that future research should enhance experimental paradigms to sufficiently increase power to conduct multilevel analyses.

Finally, STIs are typically studied in the laboratory by presenting participants with a series of isolated behavioral descriptions. This severely limits the external validity and generalizability of STI effects as currently studied. For example, the large majority of studies employed written description of behaviors. At the current point in time, we do not know whether and to what extent processing verbal behavioral information really is representative of processing directly observed behavioral information and drawing inferences about observed actors. After all, providing participants with verbal stimulus material about target persons’ behavior is a communicative act and principles of communication (e.g., Grice’s conversational maxim of relation/relevance; Grice, 1989) may influence the likelihood and/or strength of inferences drawn from that information. In a similar vein, the STI literature is largely not integrated with the broader literature of person perception (including literature from personality psychology). Future work should aim to integrate these different perspectives and methodological approaches to delineate how STIs contribute to person perception as it occurs in everyday life.

## Conclusion

To conclude, our meta-analysis showed that STIs appear to be a robust and replicable phenomenon of moderate to large effect size. Substantial heterogeneity was observed and partly explained by methodological moderators, in particular, the type of experimental paradigm. The expected moderating effect of cultural background on STIs could not be supported based on our data. Future work should seek to carefully examine relevant moderators in highly powered studies and strive for integration with other literature on person perception occurring largely in parallel.

## Supplemental Material

sj-docx-1-psp-10.1177_01461672221100336 – Supplemental material for Spontaneous Trait Inferences From Behavior: A Systematic Meta-Analysis Click here for additional data file. Supplemental material, sj-docx-1-psp-10.1177_01461672221100336 for Spontaneous Trait Inferences From Behavior: A Systematic Meta-Analysis by Antonia Bott, Larissa Brockmann, Ivo Denneberg, Espen Henken, Niclas Kuper, Felix Kruse and Juliane Degner in Personality and Social Psychology Bulletin

## Notes

1. Altogether, we contacted authors of 104 publications. Unfortunately, the requested information could often not be provided, thus limiting the generalizability of our analyses.

2. We excluded all effect sizes from the cued recall paradigm (*k* = 17) from our main analyses (see eligibility criterion 7 and Supplemental Material A). However, we report the results from the cued recall paradigm in Table S1.

3. We further included data from one Australian study. However, this study was not included in the moderator analyses because its paradigm could not be allocated to one of our paradigm categories and paradigm served as a control variable for the moderator analyses.

4. In the addendum to the preregistration, we further specified that cultural background (independent vs. interdependent) would be included in the base model if it significantly moderates the STI effect, which it did not. Thus, we deemed it most appropriate to also test effects of cultural background against the base model including paradigm.

5. In case of diverging conclusions between the approach of (1) calculating mean effect sizes for certain moderator levels and (2) the regression coefficients from the main moderator analyses, the latter should be assigned more weight given that they are model-based and control for more potential confounds (i.e., paradigm).

6. All other instructions had insufficient data with *k* < 5 or were highly confounded with paradigm type. The instruction to “respond as fast and accurately as possible” was used in 95.3% of contrasts from working memory paradigms but was never used in any of the long-term memory paradigms.

7. When only including studies that simultaneously assessed samples from independent and interdependent cultures, a significant moderation by cultural orientation was observed, with larger effect sizes in samples with independent cultural orientation (see Table S2 in the supplemental materials). However, given that only four studies providing eight effect sizes fulfilled this inclusion criterion, this result has to be interpreted with caution.

8. The transformation was based on the formulas from Lakens (2013). In particular, we multiplied *d*_z_ with the square root of 2 × (1 − *r*), with *r* indicating the correlation between participants’ responses in the two STI conditions. Correlations between responses were either coded or calculated based on *M*, *SD/SE*, and *d*_z_. Correlations were averaged within samples and an unweighted average was calculated across all samples.

## References

[bibr1-01461672221100336] Appelbaum M. Cooper H. Kline R. B. Mayo-Wilson E. Nezu A. M. Rao S. M. (2018). Journal article reporting standards for quantitative research in psychology: The APA Publications and Communications Board task force report. American Psychologist, 73, 3–25. 10.1037/amp0000191 29345484

[bibr2-01461672221100336] Bender R. Friede T. Koch A. Kuss O. Schlattmann P. Schwarzer G. Skipka G. (2018). Methods for evidence synthesis in the case of very few studies. Research Synthesis Methods, 9, 382–392. 10.1002/jrsm.1297 29504289 PMC6175308

[bibr3-01461672221100336] Carlston D. E. Skowronski J. J. (1994). Savings in the relearning of trait information as evidence for spontaneous inference generation. Journal of Personality and Social Psychology, 66, 840–856. 10.1037/0022-3514.66.5.840

[bibr4-01461672221100336] Carlston D. E. Skowronski J. J. Sparks C. (1995). Savings in relearning: II. On the formation of behavior-based trait associations and inferences. Journal of Personality and Social Psychology, 69, 420–436. 10.1037/0022-3514.69.3.429 7562389

[bibr5-01461672221100336] Carstensen A. Cao A. Gao S. Frank M. C. (2021). Investigating cross-cultural differences in reasoning, vision, and social cognition through replication. In Proceedings of the Annual Meeting of the Cognitive Science Society (Vol. 43). https://escholarship.org/uc/item/3sn0030x

[bibr6-01461672221100336] Carter E. C. Schönbrodt F. D. Gervais W. M. Hilgard J. (2019). Correcting for bias in psychology: A comparison of meta-analytic methods. Advances in Methods and Practices in Psychological Science, 2, 115–144. 10.1177/2515245919847196

[bibr7-01461672221100336] Cheung M. W. L. (2014). Modeling dependent effect sizes with three-level meta-analyses: A structural equation modeling approach. Psychological Methods, 19, 211–229. 10.1037/a0032968 23834422

[bibr8-01461672221100336] Clark H. H. (1973). The language-as-fixed-effect fallacy: A critique of language statistics in psychological research. Journal of Verbal Learning and Verbal Behavior, 12, 335–359.

[bibr9-01461672221100336] D’Agostino P. R. Beegle W. (1996). A reevaluation of the evidence for spontaneous trait inferences. Journal of Experimental Social Psychology, 32, 153–164. 10.1006/jesp.1996.0007

[bibr10-01461672221100336] Fernández-Castilla B. Jamshidi L. Declercq L. Beretvas S. N. Onghena P. Van den Noortgate W. (2020). The application of meta-analytic (multi-level) models with multiple random effects: A systematic review. Behavior Research Methods, 52, 2031–2052. 10.3758/s13428-020-01373-9 32162276

[bibr11-01461672221100336] Fiedler K. Schenck W. (2001). Spontaneous inferences from pictorially presented behaviors. Personality and Social Psychology Bulletin, 27, 1533–1546. 10.1177/01461672012711013

[bibr12-01461672221100336] Gibbons R. D. Hedeker D. R. Davis J. M. (1993). Estimation of effect size from a series of experiments involving paired comparisons. Journal of Educational Statistics, 18, 271–279. 10.3102/10769986018003271

[bibr13-01461672221100336] Goren A. Todorov A. (2009). Two faces are better than one: Eliminating false trait associations with faces. Social Cognition, 27, 222–248. 10.1521/soco.2009.27.2.222

[bibr14-01461672221100336] Grice P. (1989). Studies in the way of words. Harvard University Press.

[bibr15-01461672221100336] Ham J. Vonk R. (2003). Smart and easy: Co-occurring activation of spontaneous trait inferences and spontaneous situational inferences. Journal of Experimental Social Psychology, 39, 434–447. 10.1016/S0022-1031(03)00033-7

[bibr16-01461672221100336] Hamilton D. L. Stroessner S. J. (2021). Social cognition: Understanding people and events. SAGE.

[bibr17-01461672221100336] Hofstede G. J. (2001). Culture’s consequences: Comparing values, behaviors, institutions, and organizations across nations (2nd ed.). SAGE.

[bibr18-01461672221100336] Johannes C. (2008). The influence of stereotypes on spontaneous trait inferences (STI) in the recognition-probe-paradigm [Diploma thesis, Friedrich-Schiller-Universität, Jena, Germany].

[bibr19-01461672221100336] Judd C. M. Westfall J. Kenny D. A. (2012). Treating stimuli as a random factor in social psychology: A new and comprehensive solution to a pervasive but largely ignored problem. Journal of Personality and Social Psychology, 103, 54–69. 10.1037/a0028347 22612667

[bibr20-01461672221100336] Lakens D. (2013). Calculating and reporting effect sizes to facilitate cumulative science: A practical primer for t-tests and ANOVAs. Frontiers in Psychology, 4, 1–12. 10.3389/fpsyg.2013.00863 24324449 PMC3840331

[bibr21-01461672221100336] Lee H. Shimizu Y. Masuda T. Uleman J. S. (2017). Cultural differences in spontaneous trait and situation inferences. Journal of Cross-Cultural Psychology, 48, 627–643. 10.1177/0022022117699279

[bibr22-01461672221100336] Levordashka A. Utz S. (2017). Spontaneous trait inferences on social media. Social Psychological and Personality Science, 8, 93–101. 10.1177/1948550616663803 28123646 PMC5221722

[bibr23-01461672221100336] López-López J. A. Van den Noortgate W. Tanner-Smith E. E. Wilson S. J. Lipsey M. W. (2017). Assessing meta-regression methods for examining moderator relationships with dependent effect sizes: A Monte Carlo simulation. Research Synthesis Methods, 8, 435–450. 10.1002/jrsm.1245 28556477

[bibr24-01461672221100336] Lovakov A. Agadullina E. (2021). Empirically derived guidelines for effect size interpretation in social psychology. Manuscript accepted for publication in. European Journal of Social Psychology, 51, 485–504. 10.1002/ejsp.2752

[bibr25-01461672221100336] Markus H. R. Kitayama S. (1991). Culture and the self: Implications for cognition, emotion, and motivation. Psychological Review, 98, 224–253. 10.1037/0033-295X.98.2.224

[bibr26-01461672221100336] Markus H. R. Kitayama S. (2010). Cultures and selves: A cycle of mutual constitution. Perspectives on Psychological Science, 5, 420–430. 10.1177/1745691610375557 26162188

[bibr27-01461672221100336] Masuda T. Kitayama S. (2004). Perceiver-induced constraint and attitude attribution in Japan and the US: A case for the cultural dependence of the correspondence bias. Journal of Experimental Social Psychology, 40, 409–416. 10.1016/j.jesp.2003.08.004

[bibr28-01461672221100336] McCarthy R. J. Crouch J. L. Skowronski J. J. Milner J. S. Hiraoka R. Rutledge E. Jenkins J. (2013). Child physical abuse risk moderates spontaneously inferred traits from ambiguous child behaviors. Child Abuse & Neglect, 37, 1142–1151. 10.1016/j.chiabu.2013.05.003 23790508 PMC4091040

[bibr29-01461672221100336] Miller J. G. (1984). Culture and the development of everyday social explanation. Journal of Personality and Social Psychology, 46, 961–978. 10.1037/0022-3514.46.5.961 6737211

[bibr30-01461672221100336] Miyamoto Y. Kitayama S. (2002). Cultural variation in correspondence bias: The critical role of attitude diagnosticity of socially constrained behavior. Journal of Personality and Social Psychology, 83, 1239–1248. 10.1037/0022-3514.83.5.1239 12416925

[bibr31-01461672221100336] Miyamoto Y. Kitayama S. (2018). Cultural differences in correspondence bias are systematic and multifaceted. Advances in Methods and Practices in Psychological Science, 1, 497–498. 10.1177/2515245918817076

[bibr32-01461672221100336] Moeyaert M. Ugille M. Beretvas N. S. Ferron J. Bunuan R. Van den Noortgate W. (2017). Methods for dealing with multiple outcomes in meta-analysis: A comparison between averaging effect sizes, robust variance estimation and multilevel meta-analysis. International Journal of Social Research Methodology, 20, 559–572. 10.1080/13645579.2016.1252189

[bibr33-01461672221100336] Moher D. Shamseer L. Clarke M. Ghersi D. Liberati A. Petticrew M. …Stewart L. A. (2015). Preferred reporting items for systematic review and meta-analysis protocols (PRISMA-P) 2015 statement. Systematic Reviews, 4, Article 1. 10.1186/2046-4053-4-1 PMC4320440 25554246

[bibr34-01461672221100336] Morris M. W. Peng K. (1994). Culture and cause: American and Chinese attributions for social and physical events. Journal of Personality and Social Psychology, 67, 949–971. 10.1037/0022-3514.67.6.949

[bibr35-01461672221100336] Moskowitz G. B. (2005). Social cognition: Understanding self and others. Guilford Press.

[bibr36-01461672221100336] Na J. Kitayama S. (2011). Spontaneous trait inference is culture-specific: Behavioral and neural evidence. Psychological Science, 22, 1025–1032. 10.1177/0956797611414727 21737573

[bibr37-01461672221100336] Newman L. S. (1991). Why are traits inferred spontaneously? A developmental approach. Social Cognition, 9, 221–253. 10.1521/soco.1991.9.3.221

[bibr38-01461672221100336] Nisbett R. E. Peng K. Choi I. Norenzayan A. (2001). Culture and systems of thought: Holistic versus analytic cognition. Psychological Review, 108, 291–310. 10.1037/0033-295X.108.2.291 11381831

[bibr39-01461672221100336] Olcaysoy Okten I . (2015). Implicit goal inference and implicit trait inference: Two ways of understanding the social world [Master’s thesis, Lehigh University]. LeHigh Preserve. https://asa.lib.lehigh.edu/Record/10613001

[bibr40-01461672221100336] Open Science Collaboration. (2015). Estimating the reproducibility of psychological science. Science, 349, aac4716. 10.1126/science.aac4716 26315443

[bibr41-01461672221100336] Orghian D. Garcia-Marques L. Uleman J. S. Heinke D. (2015). A connectionist model of spontaneous trait inference and spontaneous trait transference: Do they have the same underlying processes? Social Cognition, 33, 20–66. 10.1521/soco.2015.33.1.20

[bibr42-01461672221100336] Orghian D. Smith A. Garcia-Marques L. Heinke D. (2017). Capturing spontaneous trait inference with the modified free association paradigm. Journal of Experimental Social Psychology, 73, 243–258.

[bibr43-01461672221100336] Otten S. Moskowitz G. B. (2000). Evidence for implicit evaluative in-group bias: Affect-biased spontaneous trait inference in a minimal group paradigm. Journal of Experimental Social Psychology, 36, 77–89. 10.1006/jesp.1999.1399

[bibr44-01461672221100336] Pustejovsky J. E. Rodgers M. A. (2019). Testing for funnel plot asymmetry of standardized mean differences. Research Synthesis Methods, 10, 57–71. 10.1002/jrsm.1332 30506832

[bibr45-01461672221100336] Ramos T. Garcia-Marques L. Hamilton D. (2018). Spontaneous trait inference and transference: Exploring the link between names and traits. Análise Psicológica, 36, 399–408. 10.14417/ap.1320

[bibr46-01461672221100336] R Core Team. (2018). R: A language and environment for statistical computing [Computer software]. R Foundation for Statistical Computing.

[bibr47-01461672221100336] Rosenthal R. (1991). Meta-analytic Procedures for social science research (Vol. 6). SAGE.

[bibr48-01461672221100336] Rothstein H. R. Sutton A. J. Borenstein M. (Eds.). (2005). Publication bias in meta-analysis: Prevention, assessment and adjustments. John Wiley.

[bibr49-01461672221100336] Schooler J. (2011). Unpublished results hide the decline effect: Some effects diminish when tests are repeated. Nature, 470, 437–438. 10.1038/470437a 21350443

[bibr50-01461672221100336] Shimizu Y. (2012). Spontaneous trait inferences among Japanese children and adults: A developmental approach. Asian Journal of Social Psychology, 15, 112–121.

[bibr51-01461672221100336] Shimizu Y. Lee H. Uleman J. S. (2017). Culture as automatic processes for making meaning: Spontaneous trait inferences. Journal of Experimental Social Psychology, 69, 79–85. 10.1016/j.jesp.2016.08.003

[bibr52-01461672221100336] Shimizu Y. Uleman J. S. (2021). Attention allocation is a possible mediator of cultural variations in spontaneous trait and situation inferences: Eye-tracking evidence. Journal of Experimental Social Psychology, 94, 104115. 10.1016/j.jesp.2021.104115

[bibr53-01461672221100336] Stanley T. D. Doucouliagos H. (2014). Meta-regression approximations to reduce publication selection bias. Research Synthesis Methods, 5, 60–78. 10.1002/jrsm.1095 26054026

[bibr54-01461672221100336] Todorov A. Uleman J. S. (2002). Spontaneous trait inferences are bound to actors’ faces: Evidence from a false recognition paradigm. Journal of Personality and Social Psychology, 83, 1051–1065. 10.1037/0022-3514.83.5.1051 12416911

[bibr55-01461672221100336] Todorov A. Uleman J. S. (2003). The efficiency of binding spontaneous trait inferences to actors’ faces. Journal of Experimental Social Psychology, 39, 549–562. 10.1016/S0022-1031(03)00059-3

[bibr56-01461672221100336] Todorov A. Uleman J. S. (2004). The person reference process in spontaneous trait inferences. Journal of Personality and Social Psychology, 87, 482–493. 10.1037/0022-3514.87.4.482 15491273

[bibr57-01461672221100336] Triandis H. C. (2018). Introduction: Two constructs. In Nisbett R. (Ed.), Individualism and collectivism (pp. 1–18). Routledge.

[bibr58-01461672221100336] Uleman J. S. (1987). Consciousness and control: The case of spontaneous trait inferences. Personality and Social Psychology Bulletin, 13, 337–354. 10.1177/0146167287133004

[bibr59-01461672221100336] Uleman J. S. Blader S. Todorov A. (2005). Implicit impressions. In Hassin R. Uleman J. S. Bargh J. A. (Eds.), The new unconscious (pp. 362–392). Oxford University Press.

[bibr60-01461672221100336] Uleman J. S. Hon A. Roman R. J. Moskowitz G. B. (1996). On-line evidence for spontaneous trait inferences at encoding. Personality and Social Psychology Bulletin, 22, 377–394. 10.1177/0146167296224005

[bibr61-01461672221100336] Uleman J. S. Moskowitz G. B. Roman R. J. Rhee E. (1993). Tacit, manifest, and intentional reference: How spontaneous trait inferences refer to persons. Social Cognition, 11, 321–351. 10.1521/soco.1993.11.3.321

[bibr62-01461672221100336] Uleman J. S. Newman L. S. Moskowitz G. B. (1996). People as flexible interpreters: Evidence and issues from spontaneous trait inference. In Zanna M. P. (Ed.), Advances in experimental social psychology (pp. 211–279). Academic Press.

[bibr63-01461672221100336] Uleman J. S. Rim S. Saribay S. A. Kressel L. M. (2012). Controversies, questions, and prospects for spontaneous social inferences. Social and Personality Psychology Compass, 6, 657–673. 10.1111/j.1751-9004.2012.00452.x

[bibr64-01461672221100336] Uleman J. S. Saribay S. A. (2018). Initial impressions of others. In Deaux K. Snyder M. (Eds.), Oxford handbook of personality and social psychology (2nd ed., pp. 337–366). Oxford University Press.

[bibr65-01461672221100336] Uleman J. S. Saribay S. A. Gonzalez C. M. (2008). Spontaneous inferences, implicit impressions, and implicit theories. Annual Review of Psychology, 59, 329–360. 10.1146/annurev.psych.59.103006.093707 17854284

[bibr66-01461672221100336] Van den Noortgate W. López-López J. A. Marín-Martínez F. Sánchez-Meca J . (2013). Three-level meta-analysis of dependent effect sizes. Behavior Research Methods, 45, 576–594. 10.3758/s13428-012-0261-6 23055166

[bibr67-01461672221100336] van Overwalle F. Drenth T. Marsman G . (1999). Spontaneous trait inferences: Are they linked to the actor or to the action? Personality and Social Psychology Bulletin, 25, 450–462. 10.1177/0146167299025004005

[bibr68-01461672221100336] Viechtbauer W. (2010). Conducting Meta-Analyses in R with the Metafor Package. Journal of Statistical Software, 36, 1–48. 10.18637/jss.v036.i03

[bibr69-01461672221100336] Wang M. Xia J. Yang F. (2015). Flexibility of spontaneous trait inferences: The interactive effects of mood and gender stereotypes. Social Cognition, 33, 345–358. 10.1521/soco.2015.33.4.1

[bibr70-01461672221100336] Wang M. Yan B. Yang F. Zhao Y. (2018). The development of spontaneous trait inferences about the actor and spontaneous trait transferences about the informant: Evidence from children aged 8-13 years. International Journal of Psychology, 53, 269–277. 10.1002/ijop.12367 27405877

[bibr71-01461672221100336] Wang M. Yang F. (2017). The malleability of stereotype effects on spontaneous trait inferences. Social Psychology, 48, 3–18. 10.1027/1864-9335/a000288

[bibr72-01461672221100336] Wang M. Zhao Y. Li Q. Yang F. (2016). The effects of mood on spontaneous trait inferences about the actor: Evidence from Chinese undergraduates. Scandinavian Journal of Psychology, 57, 250–255. 10.1111/sjop.12283 27005679

[bibr73-01461672221100336] Wells B. M. Skowronski J. J. Crawford M. T. Scherer C. R. Carlston D. E. (2011). Inference making and linking both require thinking: Spontaneous trait inference and spontaneous trait transference both rely on working memory capacity. Journal of Experimental Social Psychology, 47, 1116–1126. 10.1016/j.jesp.2011.05.013

[bibr74-01461672221100336] Wigboldus D. H. Sherman J. W. Franzese H. L. Knippenberg A. V. (2004). Capacity and comprehension: Spontaneous stereotyping under cognitive load. Social Cognition, 22, 292–309. 10.1521/soco.22.3.292.35967

[bibr75-01461672221100336] Winter L. Uleman J. S. (1984). When are social judgments made? Evidence for the spontaneousness of trait inferences. Journal of Personality and Social Psychology, 47, 237–252. 10.1037/0022-3514.47.2.237 6481615

[bibr76-01461672221100336] Winter L. Uleman J. S. Cunniff C. (1985). How automatic are social judgments? Journal of Personality and Social Psychology, 49, 904–917. 10.1037/0022-3514.49.4.904 4057049

[bibr77-01461672221100336] Yang F. Wang M. (2016). Do bosses and subordinates make spontaneous trait inferences equally often? The effects of power on spontaneous trait inferences. Social Cognition, 34, 271–285. 10.1521/soco.2016.34.4.2

[bibr78-01461672221100336] Zárate M. A. Uleman J. S. Voils C. I. (2001). Effects of culture and processing goals on the activation and binding of trait concepts. Social Cognition, 19, 295–323. 10.1521/soco.19.3.295.21469

[bibr79-01461672221100336] Zhang Q. Fang N. (2016). The relationship between spontaneous trait inferences and spontaneous situational inferences: A developmental approach. Social Behavior and Personality: An International Journal, 44, 569–577. 10.2224/sbp.2016.44.4.569

[bibr80-01461672221100336] Zhang Q. Wang M. (2013). The development of spontaneous trait inferences: Evidence from Chinese children. Psychological Reports, 112, 887–899. 10.2466/21.07.PR0.112.3.887-899 24245079

[bibr81-01461672221100336] Zhang Q. Wang M. (2018). The primacy-of-warmth effect on spontaneous trait inferences and the moderating role of trait valence: Evidence from Chinese undergraduates. Frontiers in Psychology, 9, Article 2148. 10.3389/fpsyg.2018.02148+ PMC6240612 30483177

