# Supplementary material for: Spontaneous Trait Inferences From Behavior: A Systematic Meta-Analysis
Source: Pers Soc Psychol Bull. 2022 Jun 24;50(1):78–102. doi: 10.1177/01461672221100336 (PMC10676050; doi:10.1177/01461672221100336)
Supplement: sj-docx-1-psp-10.1177_01461672221100336 – Supplemental material for Spontaneous Trait Inferences From Behavior: A Systematic Meta-Analysis [file sj-docx-1-psp-10.1177_01461672221100336.docx]

**Spontaneous Trait Inferences from Behavior: A Systematic Meta-analysis**

**Antonia Bott1*, Larissa Brockmann1*, Ivo Denneberg1*, Espen Henken1*, Niclas Kuper2*,
Felix Kruse1, and Juliane Degner1**

#

**Supplemental Material A: Experimental paradigms**

We identified nine different experimental STI-paradigms: (1) cued recall (Winter & Uleman, 1984), (2) savings in relearning (Carlston & Skowronski, 1994), (3) false recognition (Todorov & Uleman, 2002), (4) probe recognition (McKoon & Ratcliff, 1986; adapted by Ham & Vonk, 2003), (5) word recognition (Fiedler & Schenck, 2001; Fiedler et al., 2005), (6) lexical decision (e.g., Saribay et al., 2012; Na & Kitayama, 2011), (7) delayed recognition^1^ (e.g., D’Agostino, 1991), and (8) word stem completion^[[1]](#footnote-1)^ (e.g., Whitney & Williams-Whitney, 1990) and (9) modified free association (Orghian et al., 2017). The procedural characteristics of these various experimental paradigms offer different yet complementary advantages but also methodological restrictions for the investigation of STIs that need to be kept in mind when interpreting their outcomes. We provide a description of each included experimental paradigms and their characteristics below.

**Cued recall paradigm**

The first paradigm designed to investigate STIs was the cued recall paradigm (Winter & Uleman, 1984) which assesses the recall of trait-implying sentences with the implied traits as retrieval cues. This paradigm rests on the principle of encoding specificity (Tulving & Thomson, 1973), which states that when learning focal information along with incidentally encoded secondary information, the latter will provide an effective recall cue for the former. Thus, if individuals indeed infer a trait upon reading a behavioral statement, the implied (but not explicitly presented) trait should serve as an effective retrieval cue for the statement. Indeed, the rate of recalled sentences cued with implied-trait cues is typically higher than in no-cue condition and comparable to cues that

were actually present in the statement (Uleman, Newman, et al., 1996) – indicating that participants might have inferred the trait during encoding. Note that the validity of the cued recall paradigm has been criticized when it is based on within-participants comparisons of implied-trait cues vs. non-cues because the effect can be attributed to differential output interference rather than the spontaneous encoding of trait inferences (D’Agostino & Beegle, 1996). When applied as a within-participants design, only the comparison of implied-trait vs. other semantic cue conditions can be seen as a contrast validly capturing trait inferences. When relying on this comparison, the occurrence of trait inferences is concluded from observing that trait cues are as effective at improving recall as other semantically related cues. Because such a null-effect logic is incompatible with the effect logic of all other paradigms (i.e., improvement or impairment of task performance), we excluded the effect sizes form studies using the cued recall paradigm from the main analyses but report their analyses in Table S.1 in this supplement.

**Savings in relearning paradigm**

In the savings in relearning paradigm (Carlston & Skowronski, 1994), participants are presented with a set of actor photos paired with trait-implying behavioral statements during the encoding phase. In a second learning phase (thus re-learning), the same actor photos are paired with a trait word which was either implied versus not implied by the previous statement. In a final memory test, participants are again presented with the actor photos and are asked to recall the paired trait words. The rationale of this paradigm is based on the general principle of savings in relearning (Ebbinghaus, 1885). In particular, spontaneously inferring a trait from an actor’s behavior should facilitate learning and subsequent recall of the respective trait-actor pair. This is typically supported by better recall of implied traits versus new traits or traits implied in behavioral sentences previously paired with a different person (Uleman et al., 2012).

^1^ Universität Hamburg, Germany

^2^ Universität Bielefeld, Germany

*All five authors contributed equally to this manuscript

**Corresponding Author:**

Juliane Degner, Department of Social Psychology,

Universität Hamburg, von-Melle-Park5, 20146-Hamburg,

Germany, juliane.degner@uni-hamburg.de

**False recognition paradigm**

In the false recognition paradigm (Todorov & Uleman, 2002), participants are exposed to actor photos paired with trait-implying sentences (e.g., “Emily returned the lost wallet with all the money in it”) versus behavioral sentences which explicitly contain a trait word (e.g., “Emily is so honest that she returned the lost wallet with all the money in it”) during the encoding phase. In the subsequent recognition phase, each photo is presented together with a trait word and participants are asked to indicate whether the trait word occurred in the initial sentence corresponding to the presented person photo. It is assumed that falsely recognizing a trait as part of the previously shown sentence (i.e., an incorrect yes response to a trait that was merely implied), indicates a trait inference during encoding. Indeed, the rate of false recognitions is typically higher for implied trait words as compared to various control traits (e.g., antonyms, new traits, or traits that were implied by sentences paired with other actors; Todorov & Uleman, 2002). Besides false recognition rates, RTs for correct rejections are sometimes reported as additional exploratory analyses (e.g., Levordashka & Utz, 2017; Todorov & Uleman, 2002).

**Probe recognition paradigm**

The probe recognition paradigm (McKoon & Ratcliff, 1986; adapted by Ham & Vonk, 2003) is very similar to the False Recognition paradigm with the crucial difference that recognition is tested immediately after the encoding of each trait-implying sentence on a trial-by-trial level. Specifically, participants read a trait-implying behavioral statement immediately followed by the implied trait probe and other probe words (e.g., new traits, or objects and verbs used in the previous sentence). Their task is to decide whether the probe word was part of the previous sentence or not (Uleman et al., 1996). Typically, participants show slower RTs when correctly rejecting trait words that were implied by the sentences compared to trait words not implied by the sentences. This is interpreted as evidence that a trait was inferred during encoding. Furthermore, elevated error rates indicate that implied trait words are more frequently falsely accepted as part of the previous sentence than unrelated trait words (Ham & Vonk, 2003).

**Word recognition paradigm**

In the word recognition paradigm (Fiedler & Schenck, 2001; Fiedler et al., 2005), participants are first presented with behavioral information about an actor. Afterwards, participants see a black textbox which gradually dissolves to unveil a trait word which was either implied (e.g., caring) or not implied by the previous behavior. Participants are instructed to press a key as soon as they recognize the word and then report it. Previous work has observed shorter word recognition latencies for traits implied by the behavior compared to control conditions (e.g., Fiedler & Schenck, 2001; Fiedler et al., 2005), which is interpreted as evidence for STIs. Note that the few available studies using this paradigm also differ from other studies in that they used video or monochrome silhouette picture displaying interactive behaviors (e.g., one person’s silhouette who feeds another person’s silhouette), whereas the majority of studies using the other paradigms have relied on verbal statements describing behaviors and unsystematically included social-interactive or individual behaviors.

**Lexical decision task**

In the lexical decision task (e.g., Na & Kitayama, 2011), participants are presented with trait-implying behavioral statements along with person photos during the encoding phase. The subsequent test phase presents strings of letters that participants categorize as words or non-words, for example “hateful” versus “hartluf” (Uleman et al., 1996). Participants’ performance is typically measured in error rates or response latencies. Crucially, the words are either traits implied by the behavioral sentences or other control words (e.g., new traits or implied-other). If participants drew an actor-bound inference during the initial presentation of the trait-implying sentences, this should facilitate participants’ recognition of the implied trait word (compared to control conditions, e.g., new traits or antonyms). There are two variants of the lexical decision task used in STI research. One variant is structurally similar to the false recognition task in that participants first familiarize with behavioral statements about a number of actors (e.g., Na & Kitayama, 2011). In a separated test phase, actor photos are presented as primes before each lexical decision. It is assumed that the previously drawn trait inference is stored as part of the memory representation of the actor which later facilitates responding to implied trait words as compared to control words. We categorize this variant of the lexical decision task as part of the long-term memory-based paradigms.

The other variant is structurally similar to the probe recognition paradigm in that lexical decisions are assessed on a trial-by-trial basis immediately after the presentation of each behavioral statement (e.g., Saribay et al., 2012). If trait words are inferred during the presentation of the trait-implying sentences, their activation should lead to faster lexical decisions (i.e., recognitions of the implied trait words as a word) compared to control conditions (e.g., new traits or antonyms). We categorize this variant of the lexical decision paradigm as part of the working memory-based paradigms.

**Modified free association paradigm**

In the basic variant of the modified free association paradigm (Orghian et al., 2017), participants are presented with trait-implying statements and asked to memorize them. Immediately after each statement, participants are presented with a probe word and asked to say the first word that comes to their mind, as quickly as possible. In critical trials, the probe word is the trait implied by the statement. Participants’ response latencies are measured. The rationale of this experimental para-digm is that if a concept was semantically processed (i.e., during the trait inference from behavior), activa-tion spreads to semantically associated terms (e.g., activation of the trait “friendly” might also lead to the activation of the trait “nice”). Thus, responses to trait probes related to previously inferred trait inferences should be generated faster than responses to unrelated probes. Lower response latencies in critical trials, as compared to control trials are thus interpreted as evidence for the occurrence of spontaneous trait inferences. Orghian and collegues (2017) developed several variants of this paradigm, including delayed measure, with or without subliminal priming, and with or without the presentation of portrait pictures paired with the trait-implying statements. Currently, there are too few studies available to allow inclusion of this paradigm into the moderation analysis.

**Supplemental Material B: Deviations from the Preregistration**

**Deviations from the coding procedure**

- We originally planned to ask all authors with three or more publications in this research field for unpublished studies but did not do so due to time constraints. However, we obtained several unpublished studies by other means (e.g., through the open calls for unpublished studies or by personal communication with authors we originally contacted to obtain additional information on their published studies).
- We aimed to conduct an updated search for studies directly before submission of the meta-analysis. Due to time constraints, we refrained from doing this.
- We did not use Mendeley for data management.
- As mentioned in the first addendum, we slightly deviated from the preregistered procedure for practicing and refining the coding process. The actual practice procedure was as follows: In the first two rounds of conjoint coding practice, each of the authors coded five independent publications – always coding one study from each publication. In the third round, we re-coded the five papers from the second round. Finally, in the fourth and final round, we conjointly coded three studies from a single publication. After each practice round, divergences were discussed and the coding procedure adjusted, if necessary. We did not conduct any reliability analyses.
- Due to insufficient codable information and time constraints, we did not code the following variables (these changes were pronounced in the addendum from the 27th February, 2019): Variables concerning the stimulus material: Valence, contextual information, length of statements, stimulus content, extremity of behavior, distinctiveness of behavior, extremity of behavior; variables concerning the expectations of perceivers: Stereotype congruence, norm congruence; variables concerning participants mindset: Distance/level of abstraction, as well as a the research group associated with the publication.
- In the first addendum, we changed the names of the two clusters of paradigms (memory effects and priming effects) to long-term memory based vs. working memory based paradigms. Variants of the lexical decision task were treated as working memory paradigms when the DV followed immediately after the actor-behavior presentation on a trial-by-trial basis and as long-term memory paradigms otherwise

**Deviations from and additions to analyses plans**

- Instead of only analyzing only the individualism dimension, we exploratorily analyzed all six cultural value dimensions (Hofstede, 2001).
- Because we did not calculate any inter-rater agreement, we did not exclude moderators due to low intraclass correlations.
- We had planned to use three-level meta-analysis with the meta3 function from the metaSEM R package (Cheung, 2015). However, we have become aware of the importance of modeling dependencies at each existing level. Thus, we applied the rma.mv function from the metafor package (Viechtbauer, 2010) to fit five-level models (publications, studies, samples, contrasts, participants). Variance on the sample-level was estimated as zero and this level was thus dropped.
- We did not specify the analysis strategy concerning publication bias in the addendum. Th implementation of PET-PEESE has to be considered exploratory.
- We added the analysis of the type of contrasts although this was not mentioned in the preregistration.
- Following reviewer’s suggestions, we excluded contrasts from the cued recall paradigm from analyses (11 publications, 17 effect sizes). This was deemed necessary because the only valid STI effects in this paradigm would be expressed by null effects (contrasts using semantic cues) or lack and adequate control condition (contrasts using no cues). We report the results of our main analyses including only the cued recall paradigm in Table S1
- The specific decision rule for the inclusion or exclusion of moderators and moderator levels was not pre-registered. Thus, the decision to include moderator levels and continuous moderators with at least five available effect sizes was only made after the pre-registration.
- We examined some moderators (e.g., instruction, dependent variable) only within the subset of paradigms or single paradigms to which they applied.
- Due to insufficient data, we excluded potentially bicultural samples (e.g., self-reported ethnicity “Asian” in an interdependent country such as the US) from the culture analyses.
- Some specifics of the meta-analytic models and the presentation of results were not pre-registered (e.g., calculating average effect sizes for single moderator levels; transforming the average effect size to between-subjects Cohen’s d for comparison purposes; the specific visualization of effect sizes).

**Supplemental Material C:
References of all Studies included in the Meta-Analysis**

* References with asterisk denote studies using the cued recall paradigm, excluded from the main analyses. Their results are summarized in Table S1.

Bassili, J. N., & Smith, M. C. (1986). On the spontaneity of trait attribution: Converging evidence for the role of cognitive strategy. *Journal of Personality and Social Psychology*, *50*, 239-245.

https://doi.org/10.1037/0022-3514.50.2.239

Benrós, M. F. (2018). *Inferindo espontaneamente traços de personalidade para dois actores de um mesmo comportamento* [Master’s thesis, Universidade de Lisboa, Lisbon, Portugal]. Repositório da Universidade de Lisboa. <http://hdl.handle.net/10451/37833>

Boecking, B., & Barnhofer, T. (2014). 'She called her partner-hence she is needy': depressed patients show increased tendencies to make spontaneous trait inferences. *Psychological Medicine*, *44*, 2995-3006. https://doi.org/10.1017/S0033291714000622

Brown, R. D., & Bassili, J. N. (2002). Spontaneous trait associations and the case of the superstitious banana. *Journal of Experimental Social Psychology*, *38*, 87-92. https://doi.org/10.1006/jesp.2001.1486

Carlston, D. E., & Skowronski, J. J. (1994). Savings in the relearning of trait information as evidence for spontaneous inference generation. *Journal of Personality and Social Psychology*, *66*, 840-856. https://doi.org/10.1037/0022-3514.66.5.840

Carlston, D. E., Skowronski, J. J., & Sparks, C. (1995). Savings in relearning: II. On the formation of behavior-based trait associations and inferences. *Journal of Personality and Social Psychology*, *69*, 420-436.

https://doi.org/10.1037/0022-3514.69.3.429

Casper, C., Rothermund, K., & Wentura, D. (2011). The activation of specific facets of age stereotypes depends on individuating information. *Social Cognition*, *29*, 393-414. https://doi.org/10.1521/soco.2011.29.4.393

Costabile, K. A. (2016). Narrative construction, social perceptions, and the situation model. *Personality and Social Psychology Bulletin*, *42*, 589-602.

https://doi.org/10.1177/0146167216636627

Cowley, E. (2005). Views from consumers next in line: the fundamental attribution error in a service setting. *Journal of the Academy of Marketing Science*, *33*, 139-152. https://doi.org/10.1177/0092070304268627

Crawford, M. T., McCarthy, R. J., Kjærstad, H. L., & Skowronski, J. J. (2013). Inferences are for doing: The impact of approach and avoidance states on the generation of spontaneous trait inferences. *Personality and Social Psychology Bulletin*, *39*, 267-278. https://doi.org/10.1177/0146167212473158

Crawford, M. T., Sherman, S. J., & Hamilton, D. L. (2002). Perceived entitativity, stereotype formation, and the interchangeability of group members. *Journal of Personality and Social Psychology*, *83*, 1076-1094. https://doi.org/10.1037/0022-3514.83.5.1076

Crawford, M. T., Skowronski, J. J., & Stiff, C. (2007a). Limiting the spread of spontaneous trait transference. *Journal of Experimental Social Psychology*, *43*, 466-472. https://doi.org/10.1016/j.jesp.2006.04.003

Crawford, M. T., Skowronski, J. J., Stiff, C., & Leonards, U. (2008). Seeing, but not thinking: Limiting the spread of

spontaneous trait transference II. *Journal of Experimental Social Psychology*, *44*, 840-847. .
https://doi.org/10.1016/j.jesp.2006.04.003

Crawford, M. T., Skowronski, J. J., Stiff, C., & Scherer, C. R. (2007b). Interfering with inferential, but not associative, processes underlying spontaneous trait inference. *Personality and Social Psychology Bulletin*, *33*, 677-690. https://doi.org/10.1177/0146167206298567

*D'Agostino, P. R., & Beegle, W. (1996). A reevaluation of the evidence for spontaneous trait inferences. *Journal of Experimental Social Psychology*, *32*, 153-164.

https://doi.org/10.1006/jesp.1996.0007

D'Agostino, P. R., & Hawk, M. (1998). The transfer of actortrait associations inferred from behavior. *Social cognition, 16,* 391-399.

https://doi.org/10.1006/jesp.1996.0007

*Duff, K. J., & Newman, L. S. (1997). Individual differences in the spontaneous construal of behavior: Idiocentrism and the automatization of the trait inference process. *Social Cognition*, *15*, 217-241.

https://doi.org/10.1521/soco.1997.15.3.217

Elsbach, K. D., Cable, D. M., & Sherman, J. W. (2010). How passive ‘face time’ affects perceptions of employees: Evidence of spontaneous trait inference. *Human Relations*, *63*, 735.

https://doi.org/10.1177/0018726709353139

Fiedler, K., & Schenck, W. (2001). Spontaneous inferences from pictorially presented behaviors. *Personality and Social Psychology Bulletin*, *27*, 1533-1546.

https://doi.org/10.1177/01461672012711013

Fiedler, K., Schenck, W., Watling, M., & Menges, J. I. (2005). Priming trait inferences through pictures and moving pictures: The impact of open and closed mindsets. *Journal of Personality and Social Psychology, 88*, 229-244. https://doi.org/10.1037/0022-3514.88.2.229

Gill, M. J., & Andreychik, M. R. (2014). The Social Explanatory Styles Questionnaire: Assessing moderators of basic social-cognitive phenomena including spontaneous trait inference, the fundamental attribution error, and moral blame. *PloS one*, *9*, e100886. https://doi.org/10.1371/journal.pone.0100886

Gonzalez, C.M., Todorov, A., & Uleman, J. S. (2020). Spontaneous trait inferences from incongruent gender-stereotypic behaviors [Unpublished manuscript].

Goren, A., & Todorov, A. (2009). Two faces are better than one: Eliminating false trait associations with faces. *Social Cognition*, *27*, 222-248.

https://doi.org/10.1521/soco.2009.27.2.222

Ham, J., & Vonk, R. (2003). Smart and easy: Co-occurring activation of spontaneous trait inferences and spontaneous situational inferences. *Journal of Experimental Social Psychology*, *39*, 434-447.

https://doi.org/10.1016/S0022-1031(03)00033-7

Ham, J., & Vonk, R. (2011). Impressions of impression management: Evidence of spontaneous suspicion of ulterior motivation. *Journal of Experimental Social Psychology*, *47*, 466-471.

https://doi.org/10.1016/j.jesp.2010.12.008

Hamilton, D. L., Chen, J. M., Ko, D. M., Winczewski, L., Banerji, I., & Thurston, J. A. (2015). Sowing the seeds of stereotypes: Spontaneous inferences about groups. *Journal of Personality and Social Psychology*, *109*, 569-588.

https://doi.org/10.1037/pspa0000034

Johannes, C. (2008). *Der Einfluss von Stereotypen auf Spontaneous Trait Inferences (STI) im Recognition -Probe-Paradigma* [Diploma thesis, Friedrich-Schiller-Universität, Jena, Germany].

Kruse, F. (2017). [Unpublished raw data]. University of Hamburg, Germany.

Kruse, F., & Degner, J. (2021). Spontaneous state inferences. *Journal of Personality and Social Psychology, 121*, 774–791.

https://doi.org/10.1037/pspa0000232

Lass-Hennemann, J., Kuehl, L. K., Schulz, A., Oitzl, M. S., & Schachinger, H. (2011). Stress strengthens memory of first impressions of others' positive personality traits. *PloS one*, *6*, e16389.

https://doi.org/10.1371/journal.pone.0016389

Lee, H., Shimizu, Y., Masuda, T., & Uleman, J. S. (2017). Cultural differences in spontaneous trait and situation inferences. *Journal of Cross-Cultural Psychology*, *48*, 627-643. https://doi.org/10.1177/0022022117699279

Lee, J., & Uleman, J. S. (2020). *Comparing two paradigms for measuring spontaneous trait inferences* [Unpublished manuscript]. New York University, NY.

Levordashka, A., & Utz, S. (2017). Spontaneous trait inferences on social media. *Social Psychological and Personality Science, 8,* 93-101.

https://doi.org/10.1177/1948550616663803

*Ma, N., Vandekerckhove, M., Van Overwalle, F., Seurinck, R., & Fias, W. (2011). Spontaneous and intentional trait inferences recruit a common mentalizing network to a different degree: spontaneous inferences activate only its core areas. *Social Neuroscience*, *6*, 123-138. https://doi.org/10.1080/17470919.2010.485884

Maass, A., Colombo, A., Colombo, A., & Sherman, S. J. (2001). Inferring traits from behaviors versus behaviors from traits: The induction–deduction asymmetry. *Journal of Personality and Social Psychology*, *81*, 391-404.

https://doi.org/10.1037/0022-3514.81.3.391

Mangels, J., & Degner, J. (2022). [Unpublished raw data].

University of Hamburg, Germany. Manuscript in preparation: *The Effect of Stereotype Congruency on Spontaneous Trait Inferences from Behavior*.

Mangels, J., Stelter, M., & Degner, J. (2022). [Unpublished raw data]. University of Hamburg, Germany. Manuscript in preparation: *The Effect of Faceism on Spontaneous Trait Inferences from Behavior*.

McCall, T. C. (2011). *A hybrid paradigm for testing spontaneous trait inferences with fewer experimental trials* (Publication No. 1501797) [Master’s thesis, Purdue University]. ProQuest Dissertations & Theses Global.

McCarthy, R. J., Crouch, J. L., Skowronski, J. J., Milner, J. S., Hiraoka, R., Rutledge, E., & Jenkins, J. (2013). Child physical abuse risk moderates spontaneously inferred traits from ambiguous child behaviors. *Child Abuse & Neglect*, *37*, 1142-1151.

https://doi.org/10.1016/j.chiabu.2013.05.003 McCarthy, R. J., & Skowronski, J. J. (2011). The interplay of

controlled and automatic processing in the expression of spontaneously inferred traits: A PDP analysis. *Journal of Personality and Social Psychology*, *100*, 229-240. https://doi.org/10.1037/a0021991

McCarthy, R. J., & Skowronski, J. J. (2014). Disease avoidance cues interfere with spontaneous trait inferences. *Evolutionary Behavioral Sciences, 8*, 289-302. https://doi.org/10.1037/h0099105

McCarthy, R. J., Wells, B. M., Skowronski, J. J., & Carlston, D.E. (2018). Multiple behavior descriptions affect the acquisitions of STI & STT. *Psychological Reports*, *121*, 615-634.https://doi.org/10.1177/0033294117736317

Mc Culloch, K. C., Ferguson, M. J., Kawada, C. C., & Bargh, J. A. (2008). Taking a closer look: On the operation of nonconscious impression formation. *Journal of Experimental Social Psychology, 44,* 614-623.

https://doi.org/10.1016/j.jesp.2007.02.001

*Moskowitz, G. B. (1993b). Person organization with a memory set: are spontaneous trait inferences personality characterizations or behaviour labels? *European Journal of Personality*, *7*, 195-208.

https://doi.org/10.1002/per.2410070305

Na, J., & Kitayama, S. (2011). Spontaneous trait inference is culture-specific: Behavioral and neural evidence. *Psychological Science*, *22*, 1025-1032.

https://doi.org/10.1177/0956797611414727

Nauts, S. (2015). *Backlash for gender atypicality* [Doctoral dissertation, Radboud University Nijmegen]. Radboud Repository of the Radboud University Nijmegen.

*Newman, L. S. (1993). How individualists interpret behavior: Idiocentrism and spontaneous trait inference. *Social Cognition, 11*, 249-269.

https://doi.org/10.1521/soco.1993.11.2.243

Newman, L. S. (1991). Why are traits inferred spontaneously? A developmental approach. *Social Cognition*, *9*, 221-253.

https://doi.org/10.1521/soco.1991.9.3.221

Norman, J. B., & Chen, J. (2019, June 25). *Consequences of being unable to categorize: Racial ambiguity and spontaneous trait inferences.* PsyArXiv.

https://doi.org/10.31234/osf.io/ye7j6

Nunes, C. C. G. N. (2012). *Processos subjacentes às inferências e transferências espontâneas de traço* [Master’s thesis, Universidade de Lisboa]. Repositório da Universidade de Lisboa.

Olcaysoy Okten, I. (2015). *Implicit goal inference and implicit trait inference: Two ways of understanding the social world* [Master’s thesis, Lehigh University, PA]. LeHigh Preserve.

https://asa.lib.lehigh.edu/Record/10613001

Olcaysoy Okten, I., & Moskowitz, G. B. (2019). Spontaneous goal versus spontaneous trait inferences and explanations: How ideology shapes attributions. *European Journal of Social Psychology, 50*, 177-188. https://doi.org/10.1002/ejsp.261.

Olcaysoy Okten, I., & Moskowitz, G. B. (2020). [Unpublished raw data]. New York University, NY.

Orghian, D. (2017). *New bottles for new and old wine: New proposals for the study of spontaneous trait inferences* (Publication No. 10646579) [Doctoral dissertation, Universidade de Lisboa]. ProQuest Dissertations & Theses Global.

Orghian, D., Smith, A., Garcia-Marques, L., & Heinke, D. (2017). Capturing spontaneous trait inference with the modified free association paradigm. *Journal of Experimental Social Psychology, 73*, 243-258. https://doi.org/10.1016/j.jesp.2017.07.004

Orghian, D., Ramos, T., & Garcia-Marques, L. (2018). You are cruel even if he did it: Behavior and face processing in spontaneous trait inference and transference. *Basic and Applied Social Psychology, 40,* 104-114. https://doi.org/10.1080/01973533.2018.1436056

Orghian, D., de Almeida, F., Jacinto, S., Garcia-Marques, L., & Santos, A. S. (2019a). How your power affects my impression of you. *Personality and Social Psychology Bulletin*, *45*, 495–509.

https://doi.org/10.1177/0146167218788558

Orghian, D., Ramos, T., Garcia-Marques, L., & Uleman, J. S.

(2019b). Activation is not always inference: Word-based priming in spontaneous trait inferences. *Social Cognition, 37,* 145-173.

https://doi.org/10.1521/soco.2019.37.2.145

Otten, S., & Moskowitz, G. B. (2000). Evidence for implicit evaluative in-group bias: Affect-biased spontaneous trait inference in a minimal group paradigm. *Journal of Experimental Social Psychology*, *36*, 77-89.

https://doi.org/10.1006/jesp.1999.1399

Ramos, T. M. (2009). *A flexible view of spontaneous trait inferences* [Doctoral dissertation, Instituto Universitário de Lisboa]. Repositório do ISCTE-IUL.

dle.net/10071/2841

Ramos, T., Garcia-Marques, L., & Hamilton, D. (2018). Spontaneous trait inference and transference: Exploring the link between names and traits. *Análise Psicológica*, *36*, 399-408. https://doi.org/10.14417/ap.1320

Rim, S., Uleman, J. S., & Trope, Y. (2009). Spontaneous trait inference and construal level theory: Psychological distance increases nonconscious trait thinking. *Journal of experimental social psychology*, *45*, 1088-1097. https://doi.org/10.1016/j.jesp.2009.06.015

Rim, S., Min, K. E., Uleman, J. S., Chartrand, T. L., & Carlston,

D. E. (2013). Seeing others through rose-colored glasses: An affiliation goal and positivity bias in implicit trait impressions. *Journal of Experimental Social Psychology*, *49*, 1204-1209.

https://doi.org/10.1016/j.jesp.2013.05.007

Risavy, S. D., Komar, S., & Brown, D. J. (2010). Spontaneous trait inferences and organisational actions: The formation of organisation personality perceptions. *Canadian Journal of Behavioural Science/Revue canadienne des sciences du comportement*, *42*, 139-149. https://doi.org/10.1037/a0018039

Saribay, S., Rim, S., & Uleman, J. S. (2012). Primed self-construal, culture, and stages of impression formation. *Social Psychology, 43*, 196-204.

https://doi.org/10.1027/1864-9335/a000120

Schneid, E. D., Crawford, M. T., Skowronski, J. J., Irwin, L. M., & Carlston, D. E. (2015). Thinking about other people: Spontaneous trait inferences and spontaneous evaluations. *Social Psychology*, *46*, 24-35.

https://doi.org/10.1027/1864-9335/a000218

Shimizu, Y. (2012). Spontaneous trait inferences among Japanese children and adults: A developmental approach. *Asian Journal of Social Psychology, 15*, 112-121.

https://doi.org/10.1111/j.1467-839X.2012.01370.x

Shimizu, Y. (2017). Why are negative behaviours likely to be immediately invoked traits? The effects of valence and frequency on spontaneous trait inferences. *Asian Journal of Social Psychology, 20*, 201-210. https://doi.org/10.1111/ajsp.12183

Shimizu, Y., Lee, H., & Uleman, J. S. (2017). Culture as automatic processes for making meaning: Spontaneous trait inferences. *Journal of Experimental Social Psychology*, *69*, 79-85.

https://doi.org/10.1016/j.jesp.2016.08.003

Shimizu, Y., & Uleman, J. S. (2021). Attention allocation is a possible mediator of cultural variations in spontaneous trait and situation inferences: Eye-tracking evidence. *Journal of Experimental Social Psychology*, *94*, 104115. https://doi.org/10.1016/j.jesp.2021.104115

Todd, A. R., Molden, D. C., Ham, J., & Vonk, R. (2011). The automatic and co-occurring activation of multiple so cial inferences. *Journal of Experimental Social Psychology*, *47*, 37-49.

https://doi.org/10.1016/j.jesp.2010.08.006

Todorov, A., & Uleman, J. S. (2002). Spontaneous trait inferences are bound to actors' faces: Evidence

from a false recognition paradigm. *Journal of Personality and Social Psychology*, *83*, 1051-1065.

https://doi.org/10.1037/0022-3514.83.5.1051

Todorov, A., & Uleman, J. S. (2003). The efficiency of binding spontaneous trait inferences to actors’ faces. *Journal of Experimental Social Psychology*, *39*, 549-562. https://doi.org/10.1016/S0022-1031(03)00059-3

Uleman, J. S., Hon, A., Roman, R. J., & Moskowitz, G. B. (1996b). On-line evidence for spontaneous trait inferences at encoding. *Personality and Social Psychology Bulletin*, *22*, 377-394.

https://doi.org/10.1177/0146167296224005

*Uleman, J. S., & Moskowitz, G. B. (1994). Unintended effects of goals on unintended inferences. *Journal of Personality and Social Psychology*, *66*, 490-501. https://doi.org/10.1037/0022-3514.66.3.490

*Uleman, J. S., Moskowitz, G. B., Roman, R. J., & Rhee, E. (1993). Tacit, manifest, and intentional reference: How spontaneous trait inferences refer to persons. *Social Cognition*, *11*, 321-351.

https://doi.org/10.1521/soco.1993.11.3.321

Varnum, M. E. W., Na, J., Murata, A., & Kitayama, S. (2012). Social class differences in N400 indicate differences in spontaneous trait inference. *Journal of Experimental Psychology: General, 141,* 518–526.

https://doi.org/10.1037/a0026104

Wang, M., Yan, B., Yang, F., & Zhao, Y. (2018). The development of spontaneous trait inferences about the actor and spontaneous trait transferences about the informant: Evidence from children aged 8–13 years. *International Journal of Psychology*, *53*, 269-277. https://doi.org/10.1002/ijop.12367

Wang, M., & Yang, F. (2017). The malleability of stereotype effects on spontaneous trait inferences. *Social Psychology, 48*, 3-18.

https://doi.org/10.1027/1864-9335/a000288

Wang, M., Xia, J., & Yang, F. (2015). Flexibility of spontaneous trait inferences: The interactive effects of mood and gender stereotypes. *Social Cognition*, *33*, 345-358. https://doi.org/10.1521/soco.2015.33.4.1

Wang, M., Zhao, Y., Li, Q., & Yang, F. (2016). The effects of mood on spontaneous trait inferences about the actor: Evidence from Chinese undergraduates. *Scandinavian Journal of Psychology*, *57*, 250-255.

https://doi.org/10.1111/sjop.12283

Wells, B. M., Skowronski, J. J., Crawford, M. T., Scherer, C. R., & Carlston, D. E. (2011). Inference making and linking both require thinking: Spontaneous trait inference and spontaneous trait transference both rely on working memory capacity. *Journal of Experimental Social Psychology*, *47*, 1116-1126.

https://doi.org/10.1016/j.jesp.2011.05.013

Wentura, D., & Greve, W. (2005). Assessing the structure of self-concept: Evidence for self-defensive processes by using a sentence priming task. *Self and Identity*, *4*, 193-211. https://doi.org/10.1080/13576500444000263

Whitney, P., Waring, D. A., & Zingmark, B. (1992). Task effects on the spontaneous activation of trait concepts. *Social Cognition*, *10*, 377-396.

https://doi.org/10.1521/soco.1992.10.4.377

Wigboldus, D. H., Dijksterhuis, A., & van Knippenberg, A. (2003). When stereotypes get in the way: Stereotypes obstruct stereotype-inconsistent trait inferences. *Journal of Personality and Social Psychology*, *84*, 470-484. https://doi.org/10.1037/0022-3514.84.3.470

Wigboldus, D. H., Sherman, J. W., Franzese, H. L., & Knippenberg, A. V. (2004). Capacity and comprehension: Spontaneous stereotyping under cognitive load. *Social Cognition*, *22*, 292-309.

https://doi.org/10.1521/soco.22.3.292.35967

Wilkowski, B. M., & Robinson, M. D. (2010). Associative and spontaneous appraisal processes independently contribute to anger elicitation in daily life. *Emotion*, *10*, 181-189. https://doi.org/10.1037/a0017742

Wilkowski (2020). [Unpublished raw data]. University of Wyoming, WY.

*Winter, L., & Uleman, J. S. (1984). When are social judgments made? Evidence for the spontaneousness of trait inferences. *Journal of Personality and Social Psychology*, *47*, 237-252.

https://doi.org/10.1037/00223514.47.2.237

*Winter, L., Uleman, J. S., & Cunniff, C. (1985). How automatic are social judgments? *Journal of Personality and Social Psychology*, *49*, 904-917. https://doi.org/10.1037/0022-3514.49.4.904

Yang, F., & Wang, M. (2016). Do bosses and subordinates make spontaneous trait inferences equally often? The effects of power on spontaneous trait inferences. *Social Cognition*, *34*, 271-285. https://doi.org/10.1521/soco.2016.34.4.2

*Ybarra, O., & Stephan, W. G. (1999). Attributional orientations and the prediction of behavior: The attribution– prediction bias. *Journal of Personality and Social Psychology*, *76*, 718-727.

https://doi.org/10.1037/0022-3514.76.5.718

Zárate, M. A., Uleman, J. S., & Voils, C. I. (2001). Effects of culture and processing goals on the activation and

binding of trait concepts. *Social Cognition*, *19*, 295-323. https://doi.org/10.1521/soco.19.3.295.21469

*Zelli, A., Rowell Huesmann, L., & Cervone, D. (1995). Social inference and individual differences in aggression: Evidence for spontaneous judgments of hostility. *Aggressive Behavior*, *21*, 405-417.

https://doi.org/10.1002/1098-2337

Zengel, B., Ambler, J. K., McCarthy, R. J., & Skowronski, J. J. (2017). Spontaneous trait inference and spontaneous trait transference are both unaffected by prior evaluations of informants. *Journal of Social Psychology*, *157*, 382-387.

https://doi.org/10.1080/00224545.2016.1192099 Zhang, Q., & Fang, N. (2016). The relationship between

spontaneous trait inferences and spontaneous situational inferences: a developmental approach. *Social Behavior and Personality: An international Journal*, *44*, 569-577.

https://doi.org/10.2224/sbp.2016.44.4.569

Zhang, Q., & Wang, M. (2013). The development of spontaneous trait inferences: Evidence from Chinese children. *Psychological Reports*, *112*, 887-899.

https://doi.org/10.2466/21.07.PR0.112.3.887-899 Zhang, Q., & Wang, M. (2018). The primacy-of-warmth ef-

fect on spontaneous trait inferences and the moderating role of trait valence: Evidence from Chinese undergraduates. *Frontiers in Psychology*, *9*, 2148. https://doi.org/10.3389/fpsyg.2018.02148

### Table S1

*Results from the Cued Recall Paradigm*

| **Component** | ***k*** | **Estimate [95% CI]** | **Test** |
| --- | --- | --- | --- |
| *d_z_* |  | 0.56 [0.37, 0.74] | *Z* = 5.80, *p* < .001 |
| τ_5_ | 12 | 0.17 | χ²(1) = 0.08, *p* = .781 |
| τ_4_ | 15 | 0.00 | χ²(1) = 0.00, *p* = 1.000 |
| τ_3_ | 15 | 0.00 | χ²(1) = 0.00, *p* = 1.000 |
| τ_2_ | 17 | 0.27 | χ²(1) = 6.62, *p* = .010 |

*Note.* Shown are results from the cued recall paradigm, excluding the implied versus semantic cue contrast. _ _= variation between effect sizes within samples; τ_3_ = variation between samples within studies; τ_4_ = variation between studies within publications; τ_5_ = variation between publications. CI = confidence interval.

**Table S2**

*Effect of Culture in Studies Directly Comparing Samples from two Cultures*

| **Effect** | **Records** | **Studies** | **Samples** | **Contrasts** | | **β [95% CI]** | | **Test** | |
| --- | --- | --- | --- | --- | --- | --- | --- | --- | --- |
| Interdependent (Intercept) | 3 | 4 | 4 | 4 | 0.59 [0.33, 0.86] | |  | |  |
| Independent | 3 | 4 | 4 | 4 | 0.32 [0.14, 0.49] | | χ²(1) = 7.92, *p* = .005 | |  |

*Note.* Shown is the effect of culture in studies including samples from both independent and interdependent cultures (bicultural samples were not included). Contrary to the main moderator analyses, the effect of culture is significant here. Effects have to be interpreted with caution given the low number of samples. β = regression coefficient, CI = confidence interval.

**Table S3**

*Linear and Quadratic Effects of Year and Impact Factor (IF)*

| **Effect** | **Records** | **Studies** | **Samples** | **Contrasts** | **β [95% CI]** | **Test** |
| --- | --- | --- | --- | --- | --- | --- |
| Year: linear | 75 | 136 | 176 | 247 | -0.09 [-0.15, -0.03] | χ²(1) = 8.33, *p* = .004 |
| Year: linear | 75 | 136 | 176 | 247 | -0.14 [-0.21, -0.06] |  |
| Year: quadratic | 75 | 136 | 176 | 247 | -0.05 [-0.09, 0.00] | χ²(1) = 3.59, *p* = .058 |
| IF: linear | 62 | 115 | 149 | 205 | 0.10 [0.03, 0.16] | χ²(1) = 7.72, *p* = .005 |
| IF: linear | 62 | 115 | 149 | 205 | 0.08 [-0.01, 0.17] |  |
| IF: quadratic | 62 | 115 | 149 | 205 | 0.02 [-0.06, 0.11] | χ²(1) = 0.28, *p* = .597 |

*Note.* Shown are linear and quadratic effects of year and impact factor in the prediction of the average STI effect size. Both linear effects were significant and adding quadratic effects did not result in significant fit improvement, although the quadratic effect of year was descriptively negative. IF= Impact Factor, β = regression coefficient, CI = confidence interval.

**Table S4***Descriptive Information: Categorical Moderators*

| Variable | Sum | 1a | 1b | 2a | 2b | 2c | 3a | 3b | 3c | 4a | 4b | 5a | 5b | 5c | 6a | 6b | 6c | 7a | 7b | 7c | 8a | 8b | 9a | 9b | 10a | 10b | 11a | 11b | 12a | 12b |
| --- | --- | --- | --- | --- | --- | --- | --- | --- | --- | --- | --- | --- | --- | --- | --- | --- | --- | --- | --- | --- | --- | --- | --- | --- | --- | --- | --- | --- | --- | --- |
| 1a Culture: Independent | 204 |  |  |  |  |  |  |  |  |  |  |  |  |  |  |  |  |  |  |  |  |  |  |  |  |  |  |  |  |  |
| 1b Culture: Interdependent | 55 | – |  |  |  |  |  |  |  |  |  |  |  |  |  |  |  |  |  |  |  |  |  |  |  |  |  |  |  |  |
| 2a Instruction: Memorization | 90 | 74 | 14 |  |  |  |  |  |  |  |  |  |  |  |  |  |  |  |  |  |  |  |  |  |  |  |  |  |  |  |
| 2b Instruction: Familiarization | 62 | 47 | 13 | – |  |  |  |  |  |  |  |  |  |  |  |  |  |  |  |  |  |  |  |  |  |  |  |  |  |  |
| 2c Instruction: Impression formation | 5 | 3 | 0 | – | – |  |  |  |  |  |  |  |  |  |  |  |  |  |  |  |  |  |  |  |  |  |  |  |  |  |
| 3a Contrast: Implied vs. implied other | 148 | 99 | 43 | 71 | 18 | 0 |  |  |  |  |  |  |  |  |  |  |  |  |  |  |  |  |  |  |  |  |  |  |  |  |
| 3b Contrast: Implied vs. novel trait | 87 | 74 | 10 | 16 | 31 | 4 | – |  |  |  |  |  |  |  |  |  |  |  |  |  |  |  |  |  |  |  |  |  |  |  |
| 3c Contrast: Implied vs. antonym | 10 | 10 | 0 | 3 | 0 | 0 | – | – |  |  |  |  |  |  |  |  |  |  |  |  |  |  |  |  |  |  |  |  |  |  |
| 4a DV: Error Rates/Accuracy | 135 | 105 | 25 | 77 | 24 | 2 | 93 | 24 | 2 |  |  |  |  |  |  |  |  |  |  |  |  |  |  |  |  |  |  |  |  |  |
| 4b DV: RT | 82 | 57 | 20 | 9 | 2 | 1 | 47 | 19 | 8 | – |  |  |  |  |  |  |  |  |  |  |  |  |  |  |  |  |  |  |  |  |
| 5a Sample Type: (mostly) Students | 217 | 176 | 35 | 61 | 55 | 3 | 112 | 73 | 9 | 101 | 70 |  |  |  |  |  |  |  |  |  |  |  |  |  |  |  |  |  |  |  |
| 5b Sample Type: Adults | 26 | 24 | 0 | 20 | 1 | 0 | 20 | 5 | 0 | 19 | 7 | – |  |  |  |  |  |  |  |  |  |  |  |  |  |  |  |  |  |  |
| 5c Sample Type: Children | 21 | 1 | 20 | 6 | 6 | 0 | 14 | 7 | 0 | 11 | 4 | – | – |  |  |  |  |  |  |  |  |  |  |  |  |  |  |  |  |  |
| 6a Type of actor information: Verbal, visual | 191 | 147 | 38 | 84 | 61 | 5 | 102 | 69 | 4 | 109 | 30 | 156 | 18 | 12 |  |  |  |  |  |  |  |  |  |  |  |  |  |  |  |  |
| 6b Type of actor information: Verbal | 68 | 47 | 17 | 2 | 1 | 0 | 43 | 13 | 6 | 22 | 46 | 53 | 6 | 9 | – |  |  |  |  |  |  |  |  |  |  |  |  |  |  |  |
| 6c Type of actor information: Visual | 10 | 10 | 0 | 4 | 0 | 0 | 3 | 5 | 0 | 4 | 6 | 8 | 2 | 0 | – | – |  |  |  |  |  |  |  |  |  |  |  |  |  |  |
| 7a Type of verbal actor information: Pronoun | 113 | 93 | 15 | 30 | 51 | 2 | 42 | 53 | 3 | 51 | 16 | 97 | 8 | 6 | 104 | 9 | 0 |  |  |  |  |  |  |  |  |  |  |  |  |  |
| 7b Type of verbal actor information: Name | 77 | 60 | 16 | 43 | 6 | 0 | 61 | 12 | 1 | 55 | 22 | 62 | 8 | 7 | 55 | 22 | 0 | – |  |  |  |  |  |  |  |  |  |  |  |  |
| 7c Type of verbal actor information: Label/Profession | 19 | 15 | 2 | 1 | 0 | 0 | 15 | 4 | 0 | 9 | 10 | 14 | 4 | 0 | 1 | 18 | 0 | – | – |  |  |  |  |  |  |  |  |  |  |  |
| 8a Type of verbal behavior description: Sentence | 199 | 145 | 44 | 88 | 25 | 3 | 136 | 38 | 10 | 119 | 68 | 154 | 25 | 15 | 130 | 65 | 4 | 62 | 69 | 19 |  |  |  |  |  |  |  |  |  |  |
| 8b Type of verbal behavior description: Paragraph | 51 | 40 | 11 | 2 | 29 | 1 | 6 | 41 | 0 | 7 | 5 | 44 | 1 | 6 | 48 | 3 | 0 | 48 | 0 | 0 | – |  |  |  |  |  |  |  |  |  |
| 9a Wording of verbal behavior description: Third person | 172 | 120 | 44 | 73 | 12 | 0 | 126 | 32 | 10 | 98 | 68 | 137 | 17 | 15 | 106 | 65 | 1 | 51 | 63 | 19 | 165 | 7 |  |  |  |  |  |  |  |  |
| 9b Wording of verbal behavior description: First person | 73 | 62 | 11 | 13 | 44 | 2 | 21 | 37 | 0 | 34 | 2 | 58 | 9 | 6 | 71 | 0 | 2 | 57 | 14 | 0 | 25 | 37 | – |  |  |  |  |  |  |  |
| 10a Time interval: Filler task | 91 | 74 | 15 | 17 | 55 | 3 | 23 | 47 | 1 | 37 | 5 | 80 | 2 | 6 | 84 | 5 | 2 | 75 | 4 | 1 | 45 | 40 | 29 | 51 |  |  |  |  |  |  |
| 10b Time interval: Test phase immediately after encoding | 81 | 68 | 9 | 50 | 6 | 2 | 53 | 17 | 9 | 57 | 24 | 55 | 18 | 6 | 61 | 16 | 4 | 16 | 47 | 2 | 71 | 2 | 56 | 18 | – |  |  |  |  |  |
| 11a Time interval unit: Minutes | 56 | 39 | 15 | 13 | 36 | 2 | 14 | 26 | 0 | 34 | 0 | 47 | 2 | 6 | 53 | 3 | 0 | 45 | 2 | 1 | 39 | 14 | 25 | 27 | 56 | 0 |  |  |  |  |
| 11b Time interval unit: Days | 6 | 6 | 0 | 0 | 0 | 1 | 0 | 6 | 0 | 0 | 0 | 6 | 0 | 0 | 6 | 0 | 0 | 6 | 0 | 0 | 0 | 6 | 0 | 6 | 4 | 0 | – |  |  |  |
| 12a Publication status: Published in an academic journal | 215 | 152 | 55 | 59 | 60 | 5 | 111 | 70 | 10 | 99 | 68 | 178 | 11 | 21 | 155 | 53 | 7 | 93 | 60 | 7 | 152 | 44 | 125 | 66 | 83 | 66 | 49 | 6 |  |  |
| 12b Publication status: Not published in an academic journal | 54 | 52 | 0 | 31 | 2 | 0 | 37 | 17 | 0 | 36 | 14 | 39 | 15 | 0 | 36 | 15 | 3 | 20 | 17 | 12 | 47 | 7 | 47 | 7 | 8 | 15 | 7 | 0 | – |  |

*Note.* Shown are descriptive information for the categorical moderator variables including the overall frequency (“Sum”) and the frequency with which two moderator levels occurred simultaneously. We only included data stemming from paradigms which were included in the moderator analysis. Moreover, we only report descriptive information on moderator levels that were included in the moderator analyses. However, the number of data points with a specific moderator level may vary across different analyses (e.g., when only data from specific paradigms were analyzed) and can be examined individually based on the raw data and our analysis scripts. “–“ = not applicable because variables belonged to the same categorical moderator.

**Table S5**
*Descriptive Information: Continuous Moderators*

| Variable | *M* | *SD* | 1 | 2 | 3 | 4 | 5 | 6 | 7 | 8 | 9 | 10 | 11 | 12 | 13 | 14 |
| --- | --- | --- | --- | --- | --- | --- | --- | --- | --- | --- | --- | --- | --- | --- | --- | --- |
| 1 Power distance | 47.4 | 15.7 |  |  |  |  |  |  |  |  |  |  |  |  |  |  |
| 2 Individualism vs collectivism | 69.4 | 27.6 | -.90 |  |  |  |  |  |  |  |  |  |  |  |  |  |
| 3 Masculinity versus femininity | 61.1 | 15.8 | .11 | -.10 |  |  |  |  |  |  |  |  |  |  |  |  |
| 4 Uncertainty Avoidance | 51.6 | 18.3 | -.20 | -.11 | -.01 |  |  |  |  |  |  |  |  |  |  |  |
| 5 Long vs short term orientation | 51.3 | 28.0 | .48 | -.72 | .25 | .09 |  |  |  |  |  |  |  |  |  |  |
| 6 Indulgence versus restraint | 53.5 | 17.9 | -.78 | .95 | -.21 | -.15 | -.80 |  |  |  |  |  |  |  |  |  |
| 7 Age (in years) | 22.1 | 8.1 | -.58 | .51 | -.11 | .03 | -.25 | .38 |  |  |  |  |  |  |  |  |
| 8 Gender (% male participants) | 38.5 | 13.3 | .12 | -.06 | .26 | -.15 | .14 | -.06 | -.04 |  |  |  |  |  |  |  |
| 9 Number of traits tested | 11.4 | 8.3 | -.16 | .11 | -.17 | .09 | -.08 | .10 | .14 | -.14 |  |  |  |  |  |  |
| 10 Time Interval: Seconds | 0.9 | 1.2 | -.07 | .00 | .15 | .04 | .19 | -.10 | -.58 | .25 | -.15 |  |  |  |  |  |
| 11 Time Interval: Minutes | 9.0 | 6.5 | -.23 | .22 | .12 | -.19 | -.03 | .22 | -.50 | .46 | -.24 | N/A |  |  |  |  |
| 12 Publication Year | N/A | N/A | .39 | -.46 | .14 | .12 | .29 | -.45 | .14 | .05 | -.07 | -.21 | -.47 |  |  |  |
| 13 Impact Factor | 3.2 | 1.9 | -.53 | .53 | -.08 | -.05 | -.31 | .41 | .64 | -.09 | .04 | .08 | .13 | -.39 |  |  |
| 14 Sample Size | 67.4 | 55.8 | -.14 | .09 | .06 | .16 | -.04 | .05 | .39 | .12 | .07 | .01 | .02 | .09 | .12 |  |

*Note.* Shown are descriptive information for the continuous moderator variables including their intercorrelations. We only included data stemming from paradigms which were included in the moderator analysis. The number of data points with specific moderator realizations may vary across different analyses (e.g., when only data from specific paradigms were analyzed) and can be examined individually based on the raw data and our analysis scripts. *M* = mean, *SD* = standard deviation, N/A = not applicable.

**Table S6**

*Factor levels dropped from moderator analysis*

| **Factor levels** | | **Note** |
| --- | --- | --- |
| Experimental paradigm | |  |
|  | Delayed recognition paradigm |  |
|  | Word stem completion paradigm |  |
|  | Cued recall paradigm |  |
| Instruction during behavior encoding | |  |
|  | Focus on situation |  |
|  | Suppression |  |
|  | Concurrent cognitive load |  |
|  | As fast and accurately as possible | Restricted to WM paradigms, most of which used this instruction |
| Dependent variables | |  |
|  | Free recall of sentences |  |
|  | Recall of traits |  |
|  | Completed words |  |
|  | ERPs |  |
| Sample type | |  |
|  | Elderly people |  |
| Type of actor information | |  |
|  | Type of visual actor information |  |
| Filler task | |  |
|  | Blank screen vs. followed immediately | Could not be empirically separated |
| Culture | |  |
|  | Bicultural samples | e.g., Asian Americans |
| Publication status | |  |
|  | Submitted | Collapsed (published vs. not) |
|  | Doctoral dissertation | Collapsed (published vs. not) |
|  | Undergraduate thesis | Collapsed (published vs. not) |
|  | Grey paper | Collapsed (published vs. not) |
| *Note.* Moderator levels with less than 5 effect sizes available had to be dropped from further analyses. | | |

**Figure S1***Associations Between Effect Sizes and Year of Publication*

*******Note.* Shown is the relationship between year of publication and effect sizes (left: linear, right: quadratic). The figure was created using the regplot function from the metafor package. The line represents predicted values together with 95%-confidence intervals. The models used for prediction included dummy variables for paradigms (centered at their mean values) as further predictors. The negative quadratic trend indicates that the decline of effect sizes over time is descriptively more pronounced in more recent years. However, adding the quadratic predictor did not improve model fit significantly (*p* = .058, see Table S3).

**Figure S2***Associations Between Effect Sizes and Journal Impact Factor*

******

*Note.* Shown is the relationship between journal impact factor and effect sizes (left: linear; right: quadratic). The figure was created using the regplot function from the metafor package. The line represents predicted values together with 95%-confidence intervals. The models used for prediction included dummy variables for paradigms (centered at their mean values) as further predictors. Adding the quadratic predictor did not improve model fit significantly (*p* = .597, see Table S3).

**References**

Carlston, D. E., & Skowronski, J. J. (1994). Savings in the relearning of trait information as evidence for spontaneous inference generation. *Journal of Personality and Social Psychology*, *66*, 840-856. https://doi.org/10.1037/0022-3514.66.5.840

Cheung, M. W. L. (2015). metaSEM: An R package for metaanalysis using structural

equation modeling. *Frontiers in Psychology, 5,* 1521. https://doi:10.3389/fpsyg.2014.01521

D'Agostino, P. R. (1991). Spontaneous trait inferences: Effects of recognition instructions and subliminal priming on recognition performance. *Personality and Social Psychology Bulletin, 17*, 70-77. https://doi.org/10.1177/0146167291171011

Ebbinghaus, H. (1885). *Über das Gedächtnis: Untersuchungen zur experimentellen Psychologie*. Leipzig: Duncker & Humboldt.

Fiedler, K., & Schenck, W. (2001). Spontaneous inferences from pictorially presented behaviors. *Personality and Social Psychology Bulletin*, *27*, 1533-1546. https://doi.org/10.1177/01461672012711013

Fiedler, K., Schenck, W., Watling, M., & Menges, J. I. (2005). Priming trait inferences through pictures and moving pictures: The impact of open and closed mindsets. *Journal of Personality and Social Psychology, 88*, 229-244. https://doi.org/10.1037/0022-3514.88.2.229

Ham, J., & Vonk, R. (2003). Smart and easy: Co-occurring activation of spontaneous trait inferences and spontaneous situational inferences. *Journal of Experimental Social Psychology*, *39*, 434-447. https://doi.org/10.1016/S0022-1031(03)00033-7

Hofstede, G. J. (2001). *Culture’s consequences: Comparing values, behaviors, institutions,*

*and organizations across nations* (2nd ed.). Thousand Oaks, CA: Sage publications.

Levordashka, A., & Utz, S. (2017). Spontaneous trait inferences on social media. *Social Psychological and Personality Science, 8,* 93-101. https://doi.org/10.1177/1948550616663803

McKoon, G., & Ratcliff, R. (1986). Inferences about predictable events. *Journal of Experimental Psychology: Learning, memory, and cognition*, *12*, 82-91. https://doi.org/10.1037/0278-7393.12.1.82

Na, J., & Kitayama, S. (2011). Spontaneous trait inference is culture-specific: Behavioral and neural evidence. *Psychological Science*, *22*, 1025-1032. https://doi.org/10.1177/0956797611414727

Saribay, S., Rim, S., & Uleman, J. S. (2012). Primed self-construal, culture, and stages of impression formation. *Social Psychology, 43*, 196-204. https://doi.org/10.1027/1864-9335/a000120

Todorov, A., & Uleman, J. S. (2002). Spontaneous trait inferences are bound to actors' faces: Evidence from a false recognition paradigm. *Journal of Personality and Social Psychology*, *83*, 1051-1065. https://doi.org/10.1037/0022-3514.83.5.1051

Tulving, E., & Thomson, D. M. (1973). Encoding specificity and retrieval processes in episodic memory. *Psychological Review*, *80*, 352-373. https://doi.org/10.1037/h0020071

Uleman, J. S., Newman, L. S., & Moskowitz, G. B. (1996). People as flexible interpreters: Evidence and issues from spontaneous trait inference. In M. P. Zanna (Ed.), *Advances in Experimental Social Psychology* (pp. 211-279). San Diego, CA: Academic Press.

Uleman, J. S., Rim, S., Saribay, S., & Kressel, L. M. (2012). Controversies, questions, and prospects for spontaneous social inferences. *Social and Personality Psychology Compass*, *6*, 657-673.

https://doi.org/10.1111/j.1751-9004.2012.00452.x Viechtbauer, W. (2010). Conducting Meta-Analyses in R with

the metafor Package. *Journal of Statistical Software*, *36*, 1-48.

https://doi.org/10.18637/jss.v036.i03

Winter, L., & Uleman, J. S. (1984). When are social judgments made? Evidence for the spontaneousness of trait inferences. *Journal of Personality and Social Psychology*, *47*, 237-252.

https://doi.org/10.1037/0022-3514.47.2.237

Whitney, P., & Williams-Whitney, D. (1990). Toward a contex-

tualist view of elaborative inferences. In A. C. Graesser & G. H. Bower (Eds.), *Psychology of Learning and Motivation* (pp. 279-293). Academic Press. https://doi.org/10.1016/S0079-7421(08)60260-1

1. Only a small number of studies used this paradigm, for most of which insufficient information was available for inclusion in this meta-analysis (*k* = 2 for the word stem completion, *k* = 0 for delayed recognition). Therefore, we do not discuss them further. [↑](#footnote-ref-1)
